# Mechanisms Underlying Range of Motion Improvements Following Acute and Chronic Static Stretching: A Systematic Review, Meta-analysis and Multivariate Meta-regression

**DOI:** 10.1007/s40279-025-02204-7

**Published:** 2025-04-03

**Authors:** Lewis A. Ingram, Grant R. Tomkinson, Noah M. A. d’Unienville, Bethany Gower, Sam Gleadhill, Terry Boyle, Hunter Bennett

**Affiliations:** 1 Alliance for Research in Exercise, Nutrition and Activity (ARENA), Allied Health and Human Performance, University of South Australia, GPO Box 2471, Adelaide, SA 5001 Australia; 2 Australian Centre for Precision Health, Allied Health and Human Performance, University of South Australia, Adelaide, SA Australia

## Abstract

**Background:**

Static stretching (SS) is routinely used in sports and clinical settings to increase joint range of motion (ROM). However, the mechanisms underlying improvements in ROM remain unclear.

**Objective:**

We aimed to determine the effects of a single session (acute) and multiple sessions (chronic) of SS on stretch tolerance, passive stiffness and fascicle length, and whether such effects are moderated by specific training parameters and participant characteristics. A secondary aim was to explore the mechanisms associated with improved ROM.

**Methods:**

Seven databases (CINAHL Complete, Cochrane CENTRAL, Embase, Emcare, MEDLINE, Scopus and SPORTDiscus) were systematically searched up to 6 June, 2024. Randomised and non-randomised controlled trials investigating the effects of acute (single session) or chronic (two or more sessions) SS on muscle–tendon unit structure (fascicle length), mechanical properties (stiffness) or stretch tolerance (maximum tolerable passive resistive torque) compared to non-stretching passive controls (adults aged ≥ 18 years) were included. The effects of SS were examined using a multi-level meta-analysis, with associations between changes in maximum tolerable passive resistive torque, stiffness and fascicle length with improvements in ROM examined using multivariate meta-regression.

**Results:**

Data from 65 studies representing 1542 adults (71% male; mean ± standard deviation age = 26.1 ± 11 years) were included. We found a small decrease in overall stiffness following both acute (Hedges’ *g* = 0.42, 95% confidence interval [CI] 0.21, 0.63, *p* < 0.001) and chronic SS (Hedges’ *g* = 0.37, 95% confidence interval 0.18, 0.56, *p* < 0.001), and a moderate increase in maximum tolerable passive resistive torque following chronic SS (Hedges’ *g* = 0.74, 95% CI 0.38, 1.10, *p* < 0.001). Neither acute nor chronic SS had a significant effect on fascicle length. For acute SS, greater reductions in overall stiffness were found with moderate (*p* < 0.002) and high SS intensities (*p* = 0.02) compared with low-intensity SS, and in individuals with normal flexibility compared with those with poor flexibility at baseline (*p* < 0.001). Conversely, the effects of chronic SS on overall stiffness and maximum tolerable passive resistive torque were not moderated by stretching intensity, intervention length, baseline flexibility or sex (*p* > 0.05). Last, improved ROM following chronic SS was significantly associated with both decreased overall stiffness (*g* = 0.59, 95% CI 0.08, 1.10, *p* = 0.03) and increased maximum tolerable passive resistive torque (*g* = 0.74, 95% CI 0.41, 1.09, *p* < 0.001).

**Conclusions:**

While both acute and chronic SS reduced overall stiffness, stretch tolerance only increased following chronic SS. Neither acute nor chronic SS altered fascicle length. The effect of acute SS on reduced overall stiffness was greater when stretching at a moderate or higher intensity and in those with normal flexibility. Increased ROM was significantly associated with decreased overall stiffness and increased stretch tolerance following chronic SS. Understanding the mechanisms underlying SS will assist coaches and clinicians in deciding whether and when to prescribe SS to their athletes and patients.

**Clinical Trial Registration:**

PROSPERO CRD42023420168.

**Supplementary Information:**

The online version contains supplementary material available at 10.1007/s40279-025-02204-7.

## Key Points

**Table:** 

Evidence from randomised and non-randomised controlled trials indicates that static stretching reduces overall stiffness following a single session (acute) and multiple sessions (chronic), whereas increased stretch tolerance is only observed following multiple sessions. Static stretching alone does not appear to increase fascicle length.
Specifically, acute reductions in overall stiffness occur only when stretching at moderate or high intensities, and in those with ‘normal’ flexibility.
Conversely, the magnitude of reduced overall stiffness and increased stretch tolerance following chronic static stretching was not influenced by stretching intensity, duration of stretching intervention, sex or baseline level of flexibility.
Increased range of motion was significantly associated with both reduced overall stiffness and increased stretch tolerance.

## Introduction

Static stretching (SS) is commonly used in sporting and clinical settings to increase joint range of motion (ROM) with the intention of improving physical performance [1, 2] and reducing the risk of injury [3]. Although its role in performance and injury is controversial, [4–8] it is universally agreed that SS improves ROM [9–16]. Despite its widespread use, the physiological mechanisms underlying these increases are not well understood. Current opinion is divided over whether acute (i.e. a single bout) or chronic (i.e. long-term multiple bouts) SS generates mechanical and/or structural adaptations of the muscle–tendon unit (MTU) [13, 17–19] or whether the increased joint ROM reflects an increased tolerance to stretch [20–23].

Because of the viscoelastic properties of the MTU, there is a non-linear increase in passive torque during passive muscle lengthening [24]. Studies have used changes in passive torque following SS to infer its mechanistic effect [22, 25–29]. For example, a reduction in passive torque at a given muscle length would imply a mechanical change due to reduced passive stiffness of the MTU. Changes in stiffness have also been quantified by calculating and comparing the gradient of the torque–angle curve during passive joint movement before and after SS [22, 30, 31]. Because these methods cannot differentiate between the various tissues comprising the MTU that influence stiffness (i.e. muscles, tendons, fascia, ligaments, nerves, joint capsule) [19], recent studies have included instruments such as ultrasonic shear-wave elastography to directly measure muscle stiffness [32–35]. Ultrasonography is also commonly used following SS to quantify structural adaptations of the MTU by measuring changes in fascicle length [36–38]. Conversely, a change in stretch tolerance is assumed following SS when the ROM increases without changes in passive torque at a given submaximal muscle length, stiffness or fascicle length, or when the newly acquired ROM corresponds with increased passive torque, commonly reported as an increase in maximum tolerable passive resistive torque (PRT) [20]. These purported mechanisms may independently occur. For example, both decreased stiffness and increased stretch tolerance following SS are implied when passive torque is both lower at a given submaximal angle and increased at the newly acquired end ROM.

Systematic review and meta-analytical evidence on the mechanistic actions of SS is conflicting. Freitas et al. [20] concluded that 3–8 weeks of SS two or more times per week moderately increased the maximum tolerable PRT with no changes in stiffness or fascicle length. However, these findings were not stratified by the type of stretching modality (SS, proprioceptive neuromuscular facilitation or dynamic), making it unclear whether these results generalise to SS alone. Shah et al. [13] similarly demonstrated a moderate increase in the maximum tolerable PRT with no change in fascicle length following multiple sessions of SS over several weeks. However, unlike Freitas et al. [20], they found a reduction in muscle stiffness, suggesting that SS influenced the mechanical properties of skeletal muscle. Conversely, despite reporting no change in muscle–tendon unit (MTU) stiffness following 3–12 weeks of SS, Takeuchi et al. [18] found a moderate reduction immediately following a single session. However, in a separate meta-analysis, the same research group reported a moderate reduction in muscle stiffness following 3–12 weeks of SS [19]. In contrast to Freitas et al. [20] and Shah et al. [13], Panidi et al. [17] concluded that 3–24 weeks of SS led to trivial-to-small increases in fascicle length when measured at rest and while stretched, respectively.

Such inconsistency in findings across meta-analyses could be explained by differences in the duration, volume and intensity of stretching (i.e. training factors) or the populations studied (i.e. male, female, healthy, clinical populations, sedentary, athletic, inflexible). Only Panidi et al. [17] explored the effect of different stretching intensities, while both Panidi et al. [17] and Takeuchi et al. [18] were the only studies to investigate the influence of total stretching volume. Further investigation is needed to determine if specific training and population factors moderate SS mechanisms. Additionally, no systematic review or meta-analysis has explored whether the mechanistic causes of changes in stiffness, fascicle length and stretch tolerance are associated with improved ROM following SS. This could provide insight into which of these purported mechanisms contributes most to increases in ROM.

Better knowledge of the mechanisms underlying SS will permit more effective programming in clinical and sporting settings. For example, it has been suggested that a more compliant (i.e. less stiff) MTU following SS enhances its energy-absorbing capacity [39–41], potentially reducing the likelihood of musculotendinous injuries through better attenuation of high loads and rapid forces associated with certain activities [42]. Conversely, a less stiff MTU following SS may impair the rate of elastic recoil energy return generated during the stretch–shortening cycle, compromising performance in activities that depend on rapid stretch–shortening cycle actions [43, 44]. Understanding this will allow clinicians and coaches to decide whether and when to prescribe SS to their patients and athletes.

The primary aim of this systematic review and meta-analysis, therefore, was to investigate the effects of both a single session (acute) and multiple sessions (chronic) of SS on stiffness, fascicle length and stretch tolerance. Potential moderating variables including stretching intensity, duration, sex and baseline flexibility were also considered. The secondary aim was to explore which of these purported mechanisms are associated with improved ROM following SS.

## Methods

### Protocol and Registration

This systematic review and meta-analysis protocol was prospectively registered in the International Prospective Register of Systematic Reviews (PROSPERO) [ID: CRD42023420168] and followed the 2020 Preferred Reporting Items for Systematic reviews and Meta-Analyses (PRISMA) statement [45].

### Eligibility Criteria

This review included studies that reported at least one mechanistic outcome measure from our recent systematic review examining the effects of static stretching on flexibility [46]. We followed the PICOS (Population, Intervention, Comparison, Outcome and Study design) approach to formulate our inclusion criteria [47]:

1. *Population*: human adults aged ≥ 18 years, with no restrictions based on sex, training status, health status or baseline level of flexibility.
2. *Intervention*: SS exercise (single session [acute]) or training (multiple sessions [chronic]). Studies were excluded if they combined SS with other interventions, such as resistance training. Studies in which participants completed a warm-up after initial testing (acute) or prior to each stretching intervention session (chronic) were also excluded.
3. *Comparison*: passive (non-stretching) control group (between-subjects designs) or contralateral extremity (within-subject designs).
4. *Outcome*: pre- and post-intervention or change scores for at least one measure of stretch tolerance (maximum tolerable passive resistive torque [Nˑm], passive resistive torque at a given angle [Nˑm]), stiffness (MTU stiffness [Nˑm/°], muscle stiffness [N/mm], tendon stiffness [N/mm], shear elastic modulus [kPa] or shear wave speed [m/s]) or fascicle length [mm or cm].
5. *Study design*: randomised or non-randomised controlled trials with baseline and follow-up measures using within-subject or between-subjects study designs. Studies missing pre- and post-intervention data were excluded.
6. *Study language, publication status and timeframe*: full-text peer-reviewed journal publications written in English irrespective of publication year [48, 49]. Conference abstracts/papers, commentaries, editorials, dissertations or grey literature were excluded.

### Information Sources and Search Strategy

Seven databases (CINAHL Complete [via EBSCOhost], Cochrane CENTRAL, Embase [via Ovid], Emcare [via Ovid], MEDLINE [via Ovid], Scopus and SPORTDiscus [via EBSCOhost]) were searched on 6 June, 2024. We followed Bramer and colleagues’ [50] recommended optimal combination of databases to design the search strategy in consultation with University of South Australia academic librarians experienced in systematic literature searching. Appendix S1 of the Electronic Supplementary Material (ESM) outlines the search strategy used for each database. Further studies were identified by reviewing the reference lists of included studies and topical systematic reviews and meta-analyses [51].

### Selection Process

Records were imported into EndNote (v20.2.1; Clarivate Analytics, Philadelphia, PA, USA) and de-duplicated prior to being imported into Covidence (Veritas Health Innovation, Melbourne, VIC, Australia) for further de-duplication and record screening. All titles and abstracts were independently screened against inclusion criteria by two of the following authors (LI, HB, BG, SG and GT). The same authors, as well as ND, then independently screened full-text studies against inclusion criteria. All conflicts were resolved by majority consensus using a third author (LI for studies reviewed by HB, ND, BG, SG and GT; and HB for those reviewed by LI, ND, BG, SG and GT).

### Data Collection Process and Data Items

A single author (LI) extracted data from all included full-text studies using a custom-made standardised Excel spreadsheet (Microsoft Corporation, Redmond, WA, USA). Extracted data were verified by a second author (ND), with conflicts resolved by a third author (HB). The following data were extracted:

- lead author name and year of publication;
- article title;
- descriptive characteristics (e.g. sample size, sex, age, health status, training status, baseline level of flexibility [studies that specifically included only participants with a ROM less than a referenced average ROM were categorised as ‘poor’ flexibility, while the remaining studies were classified as either ‘average’ or ‘not reported’ [52]] for the experimental and control groups);
- region of the body and muscle group(s) stretched;
- exercise prescriptions, including duration of stretching intervention (weeks), frequency of stretching sessions (per week), number of stretches performed per session, number of repetitions per stretch, duration of each repetition (seconds) and intensity of each stretch (i.e. below the point of discomfort, until the first point of resistance or until a gentle stretch was felt [low intensity]; between discomfort and pain OR firm, noticeable tension was felt, or tightness [moderate intensity]; pain and beyond or maximal/end ROM [high intensity] [52]);
- whether stretching was supervised or unsupervised;
- whether stretching was performed unilaterally, bilaterally or both;
- participant compliance;
- study design (independent control group, crossover design or contralateral extremity used as the control);
- type of SS (active, passive, both or unclear);
- main outcomes (pre- and post-intervention means and standard deviations [SDs] or change scores) for measures of stretch tolerance, stiffness or fascicle length, along with any reported objective measures of flexibility for both the experimental and control groups.

Published means and SDs were extracted when reported, with WebPlotDigitizer (v4.6; Ankit Rohatgi, Melrose, MA, USA [http://apps.automeris.io/wpd/]) used to estimate means and SDs when presented visually [53].

### Risk of Bias Assessment

Study quality was independently assessed by two authors (LI and ND) using the Physiotherapy Evidence Database (PEDro) scale. Conflicts were resolved by a third author (HB). As in other research [12, 54–56], because it is unrealistic to blind participants and therapists in SS interventions, and that assessors are rarely blinded, we excluded items 5–7 from the 10-point PEDro scale. The methodological quality of the included studies was interpreted against an adjusted maximum PEDro score of 7 with 6–7 considered ‘excellent’, 5 ‘good’, 4 ‘moderate’ or 0–3 ‘poor’ [57].

### Certainty of Evidence

The Grading of Recommendations, Assessment, Development and Evaluation (GRADE) quality rating analysis was used by two authors (LI and HB) to independently assess the certainty of evidence [58]. Each of the three outcome measures (maximum tolerable PRT, stiffness and fascicle length) were assessed separately for acute and chronic SS studies and were categorised as either high, moderate, low or very low certainty of evidence. Because 53 of the 65 (82%) included studies were randomised controlled trials, the certainty of evidence started at high. The certainty of evidence was established by the confidence in the effect estimate and modified based on limitations in study design or execution, inconsistency of results, indirectness of evidence and imprecision. The following five criteria were used to downgrade the certainty of evidence: (i) *risk of bias* if > 25% of participants were from studies with a PEDro score < 5 out of 7 (i.e. poor or fair methodological quality) [57, 59, 60]; (ii) *inconsistency of results* if *I*^2^ > 50% (i.e. substantial or considerable heterogeneity) [60]; (iii) *indirectness* if there were significant differences in populations, outcomes or interventions used between studies; (iv) *imprecision* if data from < 800 participants per outcome were analysed [61, 62]; and (v) *publication bias* if Egger’s test was significant. Conversely, the certainty of evidence was upgraded by a single level for each of the three following criteria that were met: (i) large magnitude of effect (i.e. standardised mean difference [SMD] > 0.8); (ii) the presence of a dose–response relationship; and (iii) plausible residual opposing confounding.

### Data Synthesis and Analysis

Quantitative synthesis of data was performed with the ‘metafor’ packages in R, with plots produced using the ‘ggplot2’ package (version 4.3.1; R Core Team, https://www.r-project.org/). A multi-level meta-analysis of SMDs between conditions was conducted to examine the effects of acute and chronic SS on measures of PRT, stiffness and fascicle length, compared to non-stretching passive controls. Standardised mean differences were calculated by dividing the mean difference by the pooled SD at baseline, where the mean difference was calculated as the mean pre-post change in the SS group minus the mean pre- to post-change in the control group [63]. Where a study only reported change scores and did not report a baseline SD, the average baseline SD for all studies using the same outcome measure was used to estimate the SMD. Hedges’ g correction was applied to the SMD to adjust for potential small sample bias. In the instance where a study had multiple intervention groups, the sample size of the ‘shared’ control group was divided by the number of comparisons [64]. Similarly, when a given study had multiple outcome measures associated with the same type of outcome measure (i.e. the investigators measured muscle stiffness of a specific muscle at multiple angles), the average effect size from all measures was used for analysis. Effect sizes (g) were interpreted as trivial (< 0.20), small (0.20–0.49), moderate (0.50–0.79) and large (≥ 0.80) [65]. Positive effect sizes favoured the stretching condition, and negative effect sizes favoured the control condition. For stiffness, a positive effect size indicated that SS led to a larger reduction in stiffness than the control condition. To account for dependency between effect sizes from the same study, a multi-level random-effects model (with the study identifier as a random factor) was conducted using a restricted maximum likelihood estimation. The multi-level model was used to estimate the overall effect size and 95% confidence interval (CI).

Statistical heterogeneity between studies was assessed using *Q* and *I*^2^ statistics. *I*^2^ values were interpreted as negligible (*I*^2^ = 0–40%), moderate (*I*^2^ = 30–60%), substantial (*I*^2^ = 50–90%) or considerable (*I*^2^ = 75–100%) [66]. Subgroup analyses were conducted to explore the impact of SS on the specific structural property of the MTU that was measured (PRT at a given angle, MTU stiffness, muscle stiffness, tendon stiffness or elastic shear modulus). Given the various methods used to measure stiffness in the literature, all measures of stiffness were initially analysed collectively as ‘overall stiffness’ to determine the effect of SS on stiffness as a global construct. Subgroup analyses were then performed to identify the measures of stiffness most sensitive to SS. Potential sources of heterogeneity were also examined using the following subgroup analyses: intensity (low, moderate or high), sex (male-only, female-only or combined sex sample), baseline flexibility (poor or average), intervention duration (0–3 weeks, 4–6 weeks and > 6 weeks) for the meta-analysis of chronic SS; and intensity, sex and baseline flexibility for the meta-analysis of acute SS. For subgroup analyses, each subgroup category was included in the model as a moderator one at a time to estimate the separate effects for each subgroup (e.g. the effects of intensity were analysed independently of other subgroup categories). This was then repeated with one subgroup as the reference group to determine whether differences between subgroups were present. For all primary analyses, publication bias was inspected visually using funnel plots and examined statistically using Egger’s test. Absolute standardised residuals > 2 were considered as outliers, and sensitivity analyses were conducted whereby meta-analyses were repeated with outliers removed to determine their influence. To determine whether risk of bias influenced outcomes, a multivariate meta-regression examining the association between PEDro score and effect size estimates was also conducted to determine whether study quality influenced outcomes.

Finally, an exploratory multivariate meta-regression was conducted to examine the association between increased ROM and changes in PRT, stiffness and fascicle length to provide insight into the factors contributing most to improved flexibility. Increases in ROM were calculated using SMDs with a Hedges’ g correction, as above. For all regression analyses, the study identifier was included as a random factor to account for dependency between effect sizes from the same study. Any variables that had fewer than ten studies were not analysed as this number was considered insufficient to conduct a meta-regression [66].

## Results

### Study Selection

**Fig. 1:**
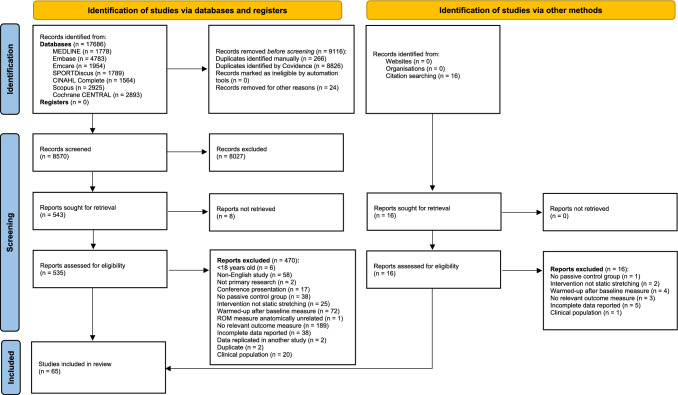
Preferred reporting items for systematic reviews and meta-analyses (PRISMA) flowchart illustrating the stages of the search and study selection process. *ROM* range of motion

A total of 17,686 studies were retrieved from the initial search. After the removal of duplicates (*n* = 9116), 8750 titles and abstracts were screened. Of these, 8027 were excluded and a further eight were unable to be retrieved, leaving 535 studies for a full-text review. From this, 65 studies were included in the final systematic review and meta-analysis [28, 29, 37, 38, 67–127]. A flow diagram of the literature search and screening process is presented in Fig. 1.

### Study Characteristics

A complete list of study characteristics can be found in Appendix S2 of the ESM. Studies included participants from 16 countries (14 high-income and two upper-middle income economies) who were examined between 1987 and 2024. Most studies (82% [*n* = 53]) randomly allocated their participants to an intervention or control group while the remaining studies (18% [*n* = 12]) used a non-randomised controlled design. Half of the studies (52% [*n* = 34]) included an independent non-stretching passive control group, 24 studies (37%) were crossover trials and seven studies (11%) used each participant’s contralateral extremity as the control.

The total number of participants was 1542 (71% male [*n* = 1098]; 29% female [*n* = 444]) with a mean ± SD participant age of 26.1 ± 11.0 years. According to the Participant Classification Framework [128], three studies (5%) comprised participants who were considered sedentary, 45% (*n* = 29) recreationally active, 11% (*n* = 7) trained, 2% (*n* = 1) athletes and 38% (*n* = 25) of unclassified training status. Ten studies (15%) included participants with predefined limitations in flexibility (i.e. ‘poor’ flexibility).

The ankle plantar flexors were the most common body region or muscle group stretched (60% [*n* = 39]), followed by the hamstrings (29% [*n* = 19]), quadriceps (8% [*n* = 5]), shoulder (2% [*n* = 1]) and hip (2% [*n* = 1]). Participants in one study stretched both their hamstrings and quadriceps. Just over half of the included studies investigated the effects of acute SS [i.e. single session] (52% [*n* = 34]), 45% (*n* = 29) investigated the effects of chronic SS (i.e. more than one session), while two studies (3%) investigated both acute and chronic effects of SS. Stretching intensity was categorised as low (8% [*n* = 5]), moderate (38% [*n* = 25]), high (40% [*n* = 26]) or not reported (11% [*n* = 7]). On average, 1.2 (± 0.7) stretching exercises were performed per session, repeated for 3.8 (± 2.7) sets. The median (interquartile range) ‘time under stretch’ was 30 (30–120) seconds per set and 3 (2–5) minutes per session. For studies investigating chronic SS, an average of 4.7 (± 2.3) sessions per week were performed over 6.5 (± 4.2) weeks. A complete list of the characteristics of the stretching interventions used in each study can be found in Appendices S3 and S4 of the ESM.

### Risk of Bias in Studies

The average methodological quality of the 65 included studies was rated as moderate based on a mean PEDro score of 4.2 (± 1.3) out of 7, with scores that range from 1 to 7. The methodological quality of nine studies (14%) was considered excellent, 11 studies (17%) good, 29 studies (45%) moderate and 16 studies (25%) poor. A lack of concealed allocation (86% [*n* = 56]), no intention-to-treat analysis (83% [*n* = 54]) and an inadequate follow-up (65% [*n* = 42]) were the most common methodological limitations. A complete list containing each study’s PEDro score is provided in Appendix S5 of the ESM.

### Synthesis of Results

#### Acute Analysis

##### Acute SS on Maximum Tolerable PRT

Acute SS had no effect on maximum tolerable PRT (*g* = 0.25, 95% CI − 0.01, 0.51, *p* = 0.05) with negligible heterogeneity between studies (Q(df = 10) = 8.1, *p* = 0.70; I^2^ = 0.0%) [refer to Appendix File 6 of the ESM]. Subgroup analyses are presented in Table 1. Effects did not differ by stretching intensity, baseline flexibility levels or sex. Inspection of funnel plots (Appendix S7 of the ESM) and the results of Egger’s test indicated no publication bias (intercept = 0.76, *p* = 0.32), and evaluation of standardised residuals identified no outliers. There was no association between PEDro score and effect size estimates (g =  − 0.09, 95% CI − 0.35, 0.18, *p* = 0.48).

**Table 1:** Subgroup analyses examining the effects of stretch intensity, baseline flexibility, sex and type of stiffness measurement on maximum tolerable PRT, stiffness and fascicle length following acute static stretching

Subgroup	*n*	Individual estimates		Between-condition comparison	
		*g* (95% CI)	*p*-value	Difference *g* (95% CI)	*p*-value
Maximum tolerable PRT					
Intensity					
Low (reference group)	1	0.45 (− 0.45, 1.36)	0.28	Reference	
Moderate	2	− 0.03 (− 0.74, 0.68)	0.92	− 0.48 (− 1.63, 0.66)	0.36
High	6	0.26 (− 0.17, 0.68)	0.20	− 0.20 (− 1.20, 0.80)	0.66
Not reported	3	0.33 (− 0.29, 0.96)	0.25	− 0.12 (− −1.22, 0.98)	0.81
Baseline flexibility					
Limited (reference group)	2	0.11 (− 0.57, 0.78)	0.73	Reference	
Normal	10	0.27 (− 0.02, 0.55)	0.06	0.16 (− 0.57, 0.90)	0.63
Sex					
Male (reference group)	5	0.10 (− 0.25, 0.46)	0.52	Reference	
Mixed	7	0.42 (0.03, 0.80)	0.04*	0.31 (− 0.22, 0.83)	0.22
Stiffness					
Stiffness measurement type					
MTU stiffness (reference)	30	0.40 (0.15, 0.66)	0.003*	Reference	
Muscle stiffness	7	0.34 (− 0.04, 0.71)	0.08	− 0.07 (− 0.45, 0.32)	0.73
Tendon stiffness	5	0.43 (0.01, 0.84)	0.045*	0.02 (− 0.39, 0.44)	0.92
PRT at a given angle	16	0.41 (0.13, 0.70)	0.006*	0.01 (− 0.30, 0.31)	0.97
Shear wave elastography	4	0.55 (0.05, 1.05)	0.03	0.15 (− 0.38, 0.68)	0.58
Intensity					
Low (reference group)	5	− 0.31 (− 0.84, 0.21)	0.30	Reference	
Moderate	24	0.65 (0.32, 0.98)	< 0.001*	0.96 (0.36, 1.56)	0.002*
High	31	0.41 (0.13, 0.70)	0.005*	0.72 (0.16, 1.29)	0.01*
Not reported	2	0.22 (− 0.71, 1.15)	0.64	0.53 (− 0.54, 1.60)	0.32
Baseline flexibility					
Limited (reference group)	4	− 0.19 (− 0.80, 0.41)	0.527	Reference	
Normal	58	0.49 (0.26, 0.71)	< 0.001*	0.68 (0.05, 1.31)	0.03*
Sex					
Male (reference group)	22	0.39 (0.05, 0.72)	0.02*	Reference	
Female	5	0.56 (− 0.27, 1.40)	0.18	0.18 (− 0.73, 1.08)	0.70
Mixed	35	0.43 (0.11, 0.75)	0.009*	0.04 (− 0.42, 0.51)	0.85
Fascicle length					
Intensity					
Moderate (reference group)	4	0.08 (− 0.61, 0.77)	0.78	Reference	
High	4	0.14 (− 0.41, 0.69)	0.56	0.06 (− 0.82, 0.94)	0.88
Baseline flexibility					
Limited (reference group)	2	0.23 (− 0.97, 1.42)	0.66	Reference	
Normal	6	0.10 (− 0.34, 0.54)	0.60	− 0.13 (− 1.4, 1.15)	0.81
Sex					
Male (reference group)	6	0.19 (− 0.32, 0.71)	0.40	Reference	
Mixed	2	− 0.04 (− 0.77, 0.69)	0.91	− 0.23 (− 1.12, 0.67)	0.56

*CI* confidence interval, *MTU* muscle–tendon unit, *PRT* passive resistive torque

*p < 0.05

##### Acute SS on Stiffness

Acute SS had a small positive effect on overall stiffness (g = 0.42, 95% CI 0.21, 0.63, *p* < 0.001) with substantial heterogeneity between studies (*Q*(*df* = 61) = 101, *p* = 0.001; *I*^2^ = 57.9%) [Appendix File 6 of the ESM]. Effects did not differ by stiffness measurement type or sex. However, overall stiffness only decreased when stretching at moderate and high intensities, and in individuals with normal flexibility at baseline. Inspection of funnel plots (Appendix File 7 of the ESM) and the results of Egger’s test indicated potential publication bias (intercept = 1.0, *p* = 0.007), and evaluation of standardised residuals identified five outliers from three studies [75, 83, 118]. Following the removal of the five outliers, the magnitude of acute SS on overall stiffness decreased (*g* = 0.33, 95% CI 0.21, 0.44, *p* < 0.001), with negligible between-study heterogeneity (*Q*(*df* = 56) = 34, *p* = 0.99; *I*^2^ = 7.2%). There was no association between PEDro score and effect size estimates (*g* = 0.06, 95% CI − 0.12, 0.24, *p* = 0.53).

##### Acute SS on Fascicle Length

Acute SS had no effect on fascicle length (*g* = 0.11, 95% CI − 0.26, 0.47, *p* = 0.52) with negligible between-study heterogeneity (*Q*(*df* = 7) = 13, *p* = 0.07; *I*^2^ = 18.9%) [Appendix File 6 of the ESM]. Effects did not differ by stretching intensity, baseline flexibility levels or sex. Inspection of funnel plots (Appendix File 7 of the ESM) and the results of Egger’s test did not indicate publication bias (intercept = 1.3, *p* = 0.30), although evaluation of standardised residuals identified one outlier [109]. Following removal of the outlier, the magnitude of acute SS on fascicle length decreased (*g* = − 0.04, 95% CI − 0.38, 0.32, *p* = 0.82), with negligible between-study heterogeneity (*Q*(*df* = 6) = 6, *p* = 0.44; *I*^2^ = 0.0%). There was no association between PEDro score and effect size estimates (*g* = 0.00, 95% CI − 0.27, 0.27, *p* = 1.00).

#### Chronic Analysis

##### Chronic SS on Maximum Tolerable PRT

Chronic SS had a moderate positive effect on maximum tolerable PRT (*g* = 0.74, 95% CI 0.38, 1.10, *p* < 0.001) with substantial heterogeneity present (*Q*(*df* = 18) = 51, *p* < 0.001; *I*^2^ = 65.4%) [Appendix File 8 of the ESM]. Subgroup analyses are presented in Table 2. Effects did not differ by intervention duration, intervention intensity, baseline flexibility levels or sex. Inspection of funnel plots (Appendix File 9 of the ESM) and the results of Egger’s test indicated high likelihood of publication bias (intercept = 2.2, *p* = 0.008), and evaluation of standardised residuals identified one outlier [71]. Following the removal of the outlier, the magnitude of chronic SS on maximum tolerable PRT increased slightly (*g* = 0.82, 95% CI 0.48, 1.16, *p* < 0.001), with moderate between-study heterogeneity (*Q*(*df* = 17) = 35, *p* = 0.06; *I*^2^ = 54.7%). There was a significant negative association between PEDro score and effect size estimates (*g* = − 0.21, 95% CI − 0.42, − 0.01, *p* = 0.04).

**Table 2:** Subgroup analyses examining the effects of duration of stretching intervention, stretch intensity, baseline flexibility, sex and type of stiffness measurement on maximum tolerable, stiffness and fascicle length following chronic static stretching

Subgroup	*n*	Individual estimates		Between-condition comparison	
		*g* (95% CI)	*p*-value	Difference *g* (95% CI)	*p*-value
Maximum tolerable PRT					
Duration, weeks					
< 4	3	0.57 (− 0.32, 1.45)	0.19	Reference	
4–6	10	0.61 (0.07, 1.14)	0.03*	0.04 (− 0.99, 1.07)	0.93
> 6	6	1.01 (0.39, 1.64)	0.004*	0.45 (− 0.53, 1.53)	0.39
Intensity					
Low (reference group)	1	0.16 (− 1.18, 1.51)	0.80	Reference	
Moderate	5	0.74 (0.12, 1.36)	0.02*	0.57 (− 0.77, 1.92)	0.38
High	9	0.47 (− 0.01, 0.96)	0.06	0.31 (− 1.12, 1.73)	0.65
Not reported	4	1.53 (0.73, 2.33)	0.001*	1.36 (− 0.20, 2.92)	0.08
Baseline flexibility					
Poor (reference group)	5	0.94 (0.26, 1.62)	0.01*	Reference	
Average/not reported	14	0.66 (0.22, 1.10)	0.006*	− 0.28 (− 1.09, 0.53)	0.53
Sex					
Male (reference group)	9	0.74 (0.24, 1.25)	0.007*	Reference	
Female	2	1.90 (0.65, 3.15)	0.005*	1.15 (− 0.20, 2.50)	0.09
Mixed	9	0.52 (0.18, 1.03)	0.04*	− 0.22 (− 0.94, 0.50)	0.52
Stiffness					
Stiffness measurement type					
MTU stiffness (reference)	12	0.40 (0.12, 0.67)	0.006*	Reference	
Muscle stiffness	8	0.36 (0.02, 0.70)	0.04*	− 0.04 (− 0.45, 0.37)	0.84
Tendon stiffness	6	0.05 (− 0.31, 0.40)	0.78	− 0.35 (− 0.77, 0.08)	0.11
PRT at a given angle	7	0.33 (0.02, 0.65)	0.04*	− 0.06 (− 0.46, 0.33)	0.75
Shear wave elastography	3	0.64 (0.17, 1.10)	0.009*	0.24 (− 0.28, 0.76)	0.35
Duration, weeks					
< 4	3	0.18 (− 0.58, 0.94)	0.63	Reference	
4–6	26	0.36 (0.13, 0.60)	0.004*	0.18 (− 0.61, 0.98)	0.64
> 6	7	0.45 (0.07, 0.83)	0.02*	0.27 (− 0.58, 1.12)	0.53
Intensity					
Low (reference group)	4	0.38 (− 0.07, 0.83)	0.10	Reference	
Moderate	11	0.14 (− 0.13, 0.41)	0.29	− 0.24 (− 0.76, 0.28)	0.36
High	17	0.42 (0.13, 0.70)	0.005*	0.03 (− 0.50, 0.57)	0.90
Not reported	4	0.82 (0.29, 1.35)	0.004*	0.44 (− 0.26, 1.14)	0.21
Baseline flexibility					
Poor (reference group)	1	0.60 (− 0.49, 1.69)	0.27	Reference	
Average/not reported	35	0.36 (0.17, 0.55)	< 0.001*	− 0.24 (− 1.34, 0.86)	0.66
Sex					
Male (reference group)	14	0.51 (0.24, 0.78)	< 0.001*	Reference	
Female	2	0.57 (− 0.27, 1.41)	0.18	0.06 (− 0.83, 0.94)	0.89
Mixed	20	0.20 (− 0.04, 0.45)	0.10	− 0.31 (− 0.68, 0.05)	0.09
Fascicle length					
Duration, weeks					
< 4	1	− 0.07 (− 1.06, 0.93)	0.89	Reference	
4–6	6	− 0.09 (− 0.45, 0.28)	0.61	− 0.02 (− 1.08, 1.04)	0.97
> 6	5	0.13 (− 0.27, 0.54)	0.48	0.20 (− 0.88, 1.27)	0.69
Intensity					
Moderate (reference group)	8	0.06 (− 0.26, 0.37)	0.69	Reference	
High	4	− 0.09 (− 0.54, 0.35)	0.65	− 0.15 (− 0.70, 0.40)	0.55
Sex					
Male (reference group)	8	− 0.06 (− 0.42, 0.29)	0.69	Reference	
Mixed	3	0.03 (− 0.36, 0.43)	0.86	0.10 (− 0.44, 0.63)	0.69

*CI* confidence interval, *MTU* muscle–tendon unit, *PRT* passive resistive torque

*p < 0.05

##### Chronic SS on Stiffness

Chronic SS had a small positive effect on overall stiffness (*g* = 0.37, 95% CI 0.18, 0.56, *p* < 0.001) with moderate heterogeneity present (*Q*(*df* = 35) = 42, *p* = 0.21; *I*^2^ = 30.0%) [Appendix File 8 of the ESM]. Effects did not differ by stiffness measurement type, intervention duration, stretch intensity, baseline flexibility levels or sex. Inspection of funnel plots (Appendix File 9 of the ESM) and the results of Egger’s test indicated potential publication bias (intercept = 1.9, *p* < 0.001), and evaluation of standardised residuals identified one outlier [90]. Following the removal of the outlier, the magnitude of chronic SS on overall stiffness remained largely unchanged (*g* = 0.37, 95% CI 0.19, 0.54, *p* < 0.001), with negligible between-study heterogeneity (*Q*(*df* = 34) = 33, *p* = 0.50; *I*^2^ = 22.3%). There was no association between PEDro score and effect size estimates (*g* = 0.02, 95% CI − 0.12, 0.16, *p* = 0.76).

##### Chronic SS on Fascicle Length

Chronic SS had no effect on fascicle length (*g* = 0.07, 95% CI − 0.25, 0.26, *p* = 0.95) with negligible heterogeneity present (*Q*(*df* = 11) = 4, *p* = 0.97; *I*^2^ = 0.0%) [Appendix File 8 of the ESM]. Effects did not differ by intervention duration, stretch intensity, baseline flexibility levels or sex. As all studies examining the effect of chronic SS on fascicle length included participants with ‘normal’ flexibility, the effect of baseline flexibility was not analysed. Inspection of funnel plots (Appendix File 9 of the ESM) and the results of Egger’s test did not indicate publication bias (intercept = 0.1, *p* = 0.74), and evaluation of standardised residuals identified no outliers. There was no association between PEDro score and effect size estimates (*g* = 0.00, 95% CI − 0.17, 0.17, *p* = 0.98).

#### Exploratory Regression Analysis

##### Association of Acute Changes in Maximum Tolerable PRT and Stiffness with Improvements in Flexibility

Of the 36 acute SS studies included in this meta-analysis, 21 (comprising 27 independent groups) examined ROM in conjunction with a maximum tolerable PRT, stiffness or fascicle length outcome. Acute SS had a moderate positive effect on ROM (*g* = 0.52, 95% CI 0.36, 0.69, *p* < 0.001) with negligible heterogeneity between studies (*Q*(*df* = 26) = 24, *p* = 0.56; *I*^2^ = 0.0%) [refer to Appendix Files 6 and 7 of the ESM, respectively].

Nine studies comprising 11 individual groups included both maximum tolerable PRT and ROM outcome measures. There was no significant association between maximum tolerable PRT and ROM (g = 0.58, 95% CI − 0.43, 1.58, *p* = 0.23) with negligible heterogeneity present (QE(*df* = 9) = 4, *p* = 0.92; *I*^2^ = 0.0%).

Eighteen studies comprising 28 individual groups and 42 measurements (muscle–tendon unit [MTU] = 21, muscle [M] = 5, tendon [T] = 4, PRT at a given angle = 10, shear wave elastography = 2) included both stiffness and ROM. There was no significant association between overall stiffness and ROM (g =  − 0.37, 95% CI − 0.87, 0.13, *p* = 0.15) with negligible heterogeneity present (QE(*df* = 40) = 37, *p* = 0.59; *I*^2^ = 30.9%). Subgroup analyses by stiffness measurement type are presented in Table 3. There was a significant association between increases in MTU stiffness and increases in flexibility, but not muscle stiffness, tendon stiffness, or PRT at a given angle. The association between changes in stiffness as measured by shear wave elastography and ROM was not explored because of an insufficient sample size (*k* = 2). Only three acute studies included measures of both fascicle length and ROM, which was not considered sufficient for meta regression.

**Table 3:** Associations between improvements in stiffness and range of motion following acute and chronic static stretching

Subgroup	*n*	Effect size estimates	
		*g* (95% CI)	*p*-value
Acute			
Stiffness measurement type			
MTU stiffness	21	− 0.73 (− 1.33, − 0.13)	0.02*
Muscle stiffness	5	− 0.45 (− 4.77, 3.88)	0.76
Tendon stiffness	4	0.19 (− 4.24, 4.62)	0.87
PRT at a given angle	10	0.13 (− 1.11, 1.37)	0.82
Chronic			
Stiffness measurement type			
MTU stiffness	11	0.44 (− 0.68, 1.57)	0.39
Muscle stiffness	8	0.48 (− 1.25, 2.21)	0.52
Tendon stiffness	6	0.29 (− 2.54, 3.11)	0.80
PRT at a given angle	7	1.57 (0.25, 2.88)	0.03*

*CI* confidence interval, *MTU* muscle–tendon unit, *PRT* passive resistive torque

*p < 0.05

##### Association of Chronic Changes in Maximum Tolerable PRT and Stiffness with Improvements in Flexibility

Of the 31 chronic studies included in this meta-analysis, 23 (comprising 25 groups) examined ROM with maximum tolerable PRT, stiffness and fascicle length outcomes. Chronic SS had a large positive effect on ROM (*g* = 0.85, 95% CI 0.56, 1.14, *p* < 0.001) with moderate heterogeneity between studies (*Q*(*df* = 24) = 59, *p* < 0.001; *I*^2^ = 61.3%) [refer to Appendix Files 8 and 9 of the ESM, respectively].

**Fig. 2:**
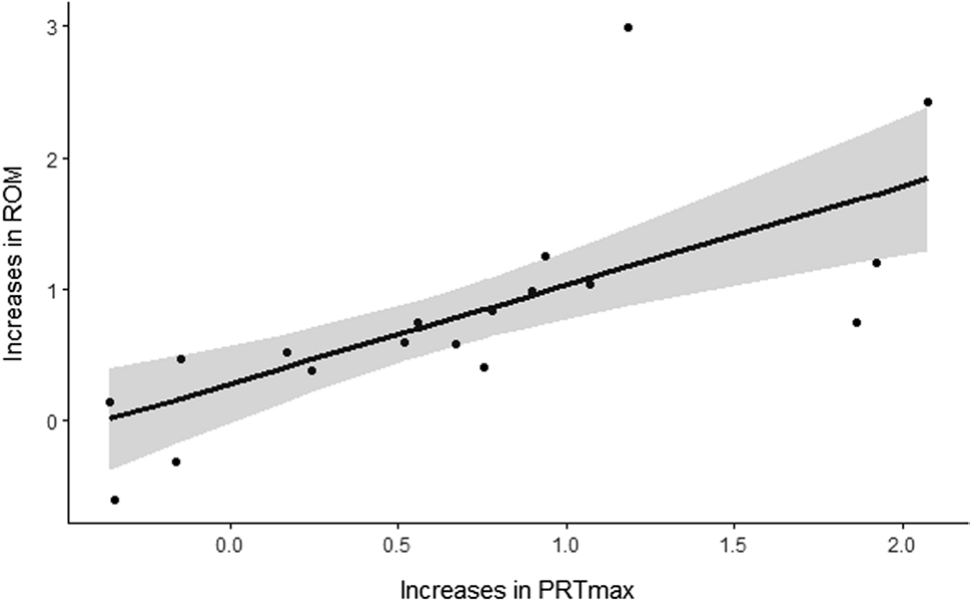
Association between increased range of motion (ROM) and increased maximum tolerable passive resistive torque (PRTmax) following chronic static stretching. The thick line represents the line of best fit. The shaded area depicts the 95% confidence interval

Sixteen studies comprising 18 individual groups included both maximum tolerable PRT and ROM outcome measures. There was a significant positive association between maximum tolerable PRT and ROM (*g* = 0.74, 95% CI 0.41, 1.09, *p* < 0.001) [Fig. 2] with negligible heterogeneity present (QE(*df* = 16) = 18, *p* = 0.33; *I*^2^ = 0.0%).

**Fig. 3:**
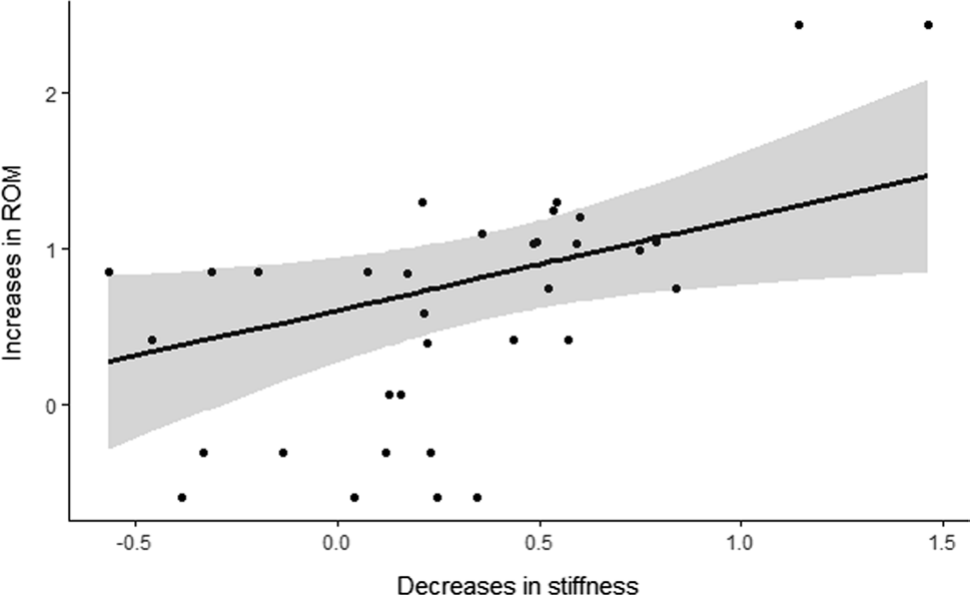
Association between increased range of motion (ROM) and decreased stiffness following chronic static stretching. The thick line represents the line of best fit. The shaded area depicts the 95% confidence interval

Seventeen studies comprising 19 groups and 34 measurements (MTU = 11, M = 8, T = 6, PRT at a given angle = 7, shear wave elastography = 2) included both stiffness and ROM outcome measures. There was a significant association between decreased overall stiffness and ROM (*g* = 0.59, 95% CI 0.08, 1.10, *p* = 0.03) [Fig. 3] with moderate between-study heterogeneity (QE(*df* = 32) = 64, *p* < 0.001; *I*^2^ = 53.8%). The subgroup analysis by stiffness measurement type is presented in Table 3. There was a significant association between increases in PRT at a given angle and increases in flexibility, but not muscle stiffness, tendon stiffness or MTU stiffness. The association between changes in stiffness as measured by shear wave ultrasound and ROM was not explored, as only two studies examined both of these outcomes. Only eight chronic studies included measures of both fascicle length and ROM, which was not considered sufficient for meta-regression.

### Certainty of Evidence

A detailed analysis of the GRADE certainty of evidence for each outcome is shown in Appendix File 10 of the ESM. The certainty of evidence across all six outcomes was downgraded one level for risk of bias; overall stiffness (acute) and maximum tolerable PRT (chronic) were both downgraded another level for inconsistency; maximum tolerable PRT (acute) and fascicle length (acute and chronic) were each downgraded an additional level for impression; and overall stiffness (acute and chronic) and maximum tolerable PRT (chronic) were further downgraded for potential publication bias. None of the outcome measures met the upgrade criteria. Therefore, the certainty of evidence was either low (maximum tolerable PRT [acute], fascicle length [acute and chronic], overall stiffness [chronic]) or very low (maximum tolerable PRT [chronic], overall stiffness [acute]).

## Discussion

Our main findings are that both acute and chronic SS reduce overall stiffness, while chronic SS increases maximum tolerable PRT. This suggests that greater tolerance to stretch may be the primary adaptation from long-term SS. No changes in fascicle length were observed following acute or chronic SS. Apart from the immediate reduction in overall stiffness, which was most pronounced when stretching at moderate or high intensities and among those with normal flexibility, none of the effects was moderated by training factors or population characteristics. Furthermore, the multi-variate meta-regression indicated that improvements in joint ROM following chronic SS are significantly associated with both a reduction in overall stiffness and an increased maximum tolerable PRT. These results suggest that longer term improvements in ROM are driven by both mechanical adaptations of the MTU as well as an increased capacity to withstand the stretching discomfort.

### Stretch Tolerance

Our findings of a moderate effect of chronic SS on maximum tolerable PRT are consistent with those of Freitas et al. [20] and Shah et al., [13] collectively suggesting increased stretch tolerance. To our knowledge, this is the first study to use meta-regression to demonstrate the positive association between improved ROM and stretch tolerance, supporting the view that chronic SS reduces the perceived discomfort when stretching towards the end of range. In contrast, while we found no change in maximal tolerable PRT following acute SS, Shah et al. [13] reported a moderate increase suggesting that stretch tolerance contributes to the immediate response. This conflict could be attributed to methodological differences between the two meta-analyses. For example, Shah et al. [13] acknowledged that their search strategy was not extensive, resulting in five effects from three studies for the effect of acute SS on maximum tolerable PRT compared with 11 effects from eight studies in our meta-analysis.

Despite this, the mechanisms underlying changes in stretch tolerance are poorly understood. One theory is that chronic SS reduces the sensitivity of the nociceptive nerve endings that innervate the MTU, resulting in a higher pain threshold and pain tolerance [129]. This may then allow the individual to push through more discomfort as they move closer towards their end ROM. The acute ischemic compression associated with specific SS positions, which has a known analgesic effect, has been postulated as another possible contributor to greater stretch tolerance [130]. However, it is plausible that neural mechanisms may best explain the changes in stretch tolerance observed following chronic SS. Guissard and Duchatea [24] suggest that in contrast to strength training, neural adaptations to SS proceed the immediate mechanical responses. Specifically, they reported a significant progressive decline in the amplitude of the H- and T-reflexes (measures of Ia afferent and muscle spindle sensitivity, respectively [131]) over the course of a 6-week training program comprising 10 min of daily ankle plantar flexor SS, which were not correlated with the reduction in passive stiffness observed at the MTU [27]. Our findings that reduced stiffness contribute most to acute improvements in ROM while greater tolerance to SS largely accounts for the chronic improvements lend support to Guissard and Duchatea [24, 27] if a neural basis for stretch tolerance is assumed. This proposed latent onset of neural adaptations to SS is further supported by Shah et al. [13], who were unable to show any effect on motor-evoked potential amplitude, H-reflex amplitude or maximum M-wave amplitude following acute SS in their meta-analysis. However, because they did not report on changes in T-reflex activity, reduced muscle spindle sensitivity cannot be ruled out as a potential neural mechanism underlying changes in stretch tolerance. Future studies are necessary to investigate neural adaptations following chronic SS and to explore their relative contribution towards an increased tolerance to stretch.

### Stiffness

Our results support the notion that SS is a sufficient stimulus to induce a small mechanical change at the MTU by reducing stiffness. While the small effect size was consistent for both acute and chronic SS, our meta-regression revealed a strong association with ROM improvements following only chronic SS. This suggests that the relative contribution of stiffness to improved ROM may play an increasingly larger role over time. Unlike acute SS, where small-to-moderate reductions in stiffness have been reported in other meta-analyses [13, 18], the effect of chronic SS on stiffness remains contentious. Indeed, both Freitas et al. [20] and Takeuchi et al. [18] did not report a change in MTU stiffness following chronic SS. As discussed previously, Freitas et al. [20] included studies on proprioceptive neuromuscular facilitation and dynamic stretching in their meta-analysis, which may have influenced their overall effect, while Takeuchi et al. [18] reported a moderate non-statistically significant effect on MTU stiffness. A key methodological difference to these meta-analyses is our exclusion of studies that included a warm-up to control for a warm-up induced reduction in soft-tissue viscoelasticity associated with heat produced by muscle contraction [132], which alone is sufficient to temporarily increase ROM and potentially reduce stiffness [4].

Another plausible reason for the discrepancy across studies could be the differences in how MTU stiffness was calculated. The common method involves calculating the gradient of the torque–angle curve as the joint is passively moved towards the end of its range [22, 31]. Inconsistency arises in how the start of the slope is defined [18]. For example, some studies identify the start of the slope as the position at which the muscle being tested is free of slack (i.e. the beginning of the linear region). This will tend to result in a steeper gradient (i.e. greater stiffness) compared with studies that measure the slope across the joint’s full ROM, which will also capture the toe region where the muscle fibres are crimped, and passive torque is minimal [133, 134]. However, the key limitation of this method is its inability to distinguish the relative contributions to stiffness provided by each of the structures that comprise the MTU. Nevertheless, our results are consistent with Warneke et al.’s [135] recent systematic and meta-analysis. They attributed their partially different findings to those of Freitas et al. [20] and Takeuchi et al. [18] to the inclusion of a larger number of recent studies and differences in meta-analytical calculation methods, respectively.

While our analysis involved pooling all measures of stiffness to summarise the overall effect of SS on stiffness, subgroup analyses found no significant differences among different measures of stiffness including MTU, muscle and tendon stiffness. However, the lack of significant differences may be in part owing to limited statistical power caused by the small number of studies, and the subgroup effect size estimates indicated that the effect of SS on tendon stiffness was notably smaller than for all other measures. Similarly, Shah et al. [13] found that chronic SS had no effect on tendon stiffness but reported both a small and moderate reduction in muscle stiffness and shear elastic modulus, respectively. These findings were further supported by Takeuchi et al. [19]. Given the difference in collagen composition between skeletal muscle tissue (1–10%) and tendons (65–80%) [136], the tendon is inherently less compliant and a greater degree of torque is required to elongate it a given length compared with muscle. Furthermore, as muscle and tendon are connected in series along the MTU, the constant external passive torque induced during stretching will subject the muscle fascicles to greater strain than the tendon [137], potentially explaining why the mechanical properties of muscle appear more responsive to SS for any given stimulus compared with the tendon. The results of our regression analysis further support this finding, with acute changes in MTU stiffness (but not tendon stiffness) significantly associated with acute changes in ROM. This underscores the importance of isolating the various structures within the MTU when assessing changes in stiffness.

Although acute SS at higher intensities may not provide any additional benefits to ROM, our results suggest that stretching to either the point of discomfort (moderate intensity) or pain (high intensity) will lead to significantly larger reductions in overall stiffness when compared with stretching below the point of discomfort (low intensity), which had no effect on overall stiffness. This is supported in a recent study by Hatano et al. [29] who reported a significantly greater reduction in MTU stiffness following 5 min of hamstring SS at an intensity *above* the onset of pain compared with *at* the onset of pain. This suggests that a minimum amount of stress must be placed on the tissues to elicit an immediate mechanical response at the MTU. If it is assumed that those with normal flexibility have had greater exposure to stretch training, they may be better conditioned to tolerate the discomfort of stretching at moderate-to-high intensities, reducing stiffness more effectively than those with poor flexibility. This could partially explain why our subgroup analysis found a larger effect for stiffness in those with normal flexibility compared with those with poor flexibility. Nevertheless, this finding needs to be interpreted with caution as only a small number of studies (*n* = 4) sampled adults with poor flexibility.

### Fascicle Length

Panidi et al. [17] recently published the first meta-analysis demonstrating sarcomerogenesis outside of animal models by providing evidence in humans for an increase in fascicle length following chronic SS. Specifically, their subgroup analysis revealed that stretching at high volumes (i.e. > 90 min) and/or high intensities (i.e. into discomfort or pain) was necessary to elicit structural adaptations at the MTU. Our findings do not support this, and nor do those previously published by Freitas et al. [20] and Shah et al. [13]. This conflict is unlikely to be explained by differences in dosage parameters, with half of the studies included in our analysis using similarly high stretching volumes (≥ 90 min), while all studies stretched at either moderate or high intensities. It should be noted that the meta-analysis by Panidi et al. [17] stratified effects by region of the same muscle (i.e. distal, medial, low and high regions of the gastrocnemius medialis) or immediately adjacent muscles (i.e. gastrocnemius medialis, gastrocnemius lateralis and soleus) obtained from multiple single studies, and did not account for within-study clustering, which may have inflated the magnitude of their summary effects [138].

This is not to suggest that sarcomerogenesis cannot occur in humans. Indeed, several studies have demonstrated increased fascicle length in response to eccentric resistance training [139–141]. Nevertheless, given that the protocol length of the studies included in our chronic SS analysis ranged from 1 to 24 weeks (average of 6 weeks), it is plausible that such durations are insufficient to induce structural adaptations. It is commonly reported in resistance training studies that interventions lasting at least 8–12 weeks are necessary to induce muscle hypertrophy, [142] so it is not unreasonable to propose that a specific minimal duration to increase the fascicle length protocol may be required to observe a homologous structural change following SS. Future studies examining changes in fascicle length over sufficiently longer periods are required to provide further insight into whether SS can sufficiently stimulate a structural change in the MTU.

### Limitations

While we were able to perform subgroup analyses for intensity, duration, baseline flexibility, and biological sex, we were unable to explore other potential moderators of interest such as specific muscle stretched, training status and age because of an insufficient number of studies. The effect of these variables remains to be examined. Of note was the lack of studies investigating female-only cohorts—a broader social issue not limited to rehabilitation and performance research [143]. This only allowed us to compare studies of male-only cohorts to mixed male and female cohorts for three of the six variables of interest. Furthermore, for those variables that we were able to separate for male and female individuals, the highest number of female individual-only studies was three (chronic SS on stiffness). Further research is needed to investigate the mechanistic effects of SS in female individuals to determine if they respond differently to male individuals. Likewise, comparisons between muscle groups were not feasible as most studies investigated either the hamstrings or ankle plantar flexors, with only six studies examining the quadriceps, and one each for the hip and shoulder. Additionally, although we were able to compare the effect of different stretching intensities, it must be noted that our attempts to classify studies into low, moderate or high intensity were based on each study’s qualitative description of the participants’ perceived level of discomfort or pain rather than an objective standardised method. This reflects a limitation inherent to most flexibility-based research in that there is currently no agreed upon method of quantifying intensity. While the current study focuses on the responses and adaptations at the level of the MTU, we did not consider neural mechanisms such as H-reflex and M-wave amplitudes that may play a key role in determining the improvements in ROM following SS. Last, we must caveat our findings given the low and very low certainty of evidence as suggested by our GRADE analysis. This reflects the need for further higher quality, primary research studies with larger sample sizes to continue to investigate the mechanisms underlying the ROM improvements following both acute and chronic SS. Finally, while we conducted a subgroup analysis to determine which aspects of stiffness contributed to the largest changes in ROM, there were few studies that reported on measures of muscle and tendon stiffness alone. As such, results of the meta-regression should be interpreted with caution. Future research should consider including multiple measures of stiffness in response to stretching to provide further insight into the mechanisms underlying increases in flexibility.

### Implications

The findings of this meta-analysis indicate that the immediate improvements in ROM following acute SS are predominantly mediated by a reduction in overall stiffness, whereas longer term adaptations appear to be driven more by a greater tolerance to stretch. Understanding the mechanisms underlying SS can inform coaches when to prescribe SS with consideration of the physical qualities required for successful performance in their specific sport. For example, an immediate increase in ROM will likely exceed the cost of a concurrent reduction in stiffness in a sport that demands high levels of flexibility such as gymnastics. Conversely, in sports for which plyometric attributes are the key to success, the risk of compromising performance by attenuating the rate of elastic recoil energy return will likely override the immediate benefit of improving ROM. In this case, programming SS after training and competition would be rational if the athlete aims to improve their ROM longer term. For the clinician treating a joint contracture following a period of prolonged immobilisation, knowing that the longer term improvements in ROM are largely attributable to increased stretch tolerance may lead them to consider another intervention such as eccentric resistance training if their goal is to increase muscle fascicle length.

## Conclusions

Both acute and chronic SS led to a small reduction in overall stiffness, while chronic SS led to a moderate increase in stretch tolerance. At present, there is insufficient evidence to suggest that up to 24 weeks of moderate- and high-intensity SS increases muscle fascicle length. While greater reductions in overall stiffness were observed with moderate- and high- intensity SS and in those with normal flexibility following acute SS, no other effects were moderated by dosage parameters or participant demographics. Improvements in ROM following chronic SS were associated with both decreased overall stiffness and increased stretch tolerance, indicating that both mechanical adaptations and a greater capacity to withstand the discomfort associated with stretching drive the long-term adaptations to SS. This information can be used by clinicians and coaches to better inform decision making regarding whether and when to prescribe SS to their patients and athletes.

## Supplementary Information

Below is the link to the electronic supplementary material.

Supplementary file1 (DOCX 27 KB)

Supplementary file2 (DOCX 60 KB)

Supplementary file3 (DOCX 46 KB)

Supplementary file4 (DOCX 29 KB)

Supplementary file5 (DOCX 30 KB)

Supplementary file6 (DOCX 38 KB)

Supplementary file7 (DOCX 7297 KB)

Supplementary file8 (DOCX 5161 KB)

Supplementary file9 (DOCX 6321 KB)

Supplementary file10 (DOCX 4386 KB)

## Declarations

### Availability of Data and Material

The data and materials necessary to reproduce the findings reported in this article are available at https://osf.io/c9kt2/.
