# Supplementary material for: Mechanisms Underlying Range of Motion Improvements Following Acute and Chronic Static Stretching: A Systematic Review, Meta-analysis and Multivariate Meta-regression
Source: Sports Med. 2025 Apr 3;55(6):1449–66. doi: 10.1007/s40279-025-02204-7 (PMC12152101; doi:10.1007/s40279-025-02204-7)
Supplement: Supplementary file 1 — Supplementary file1 (DOCX 27 KB) [file 40279_2025_2204_MOESM1_ESM.docx]

**Title:** Mechanisms Underlying Range of Motion Improvements Following Acute and Chronic Static Stretching: A Systematic Review, Meta-Analysis, and Multivariate Meta-Regression

**Journal Name:** Sports Medicine

**Authors:** Lewis Ingram^1^, Grant Tomkinson^1^, Noah D’Unienville^1^, Bethany Gower^1^, Sam Gleadhill^1^, Terry Boyle^2^, and Hunter Bennett^1^

**Affiliations:**

^1^Alliance for Research in Exercise, Nutrition and Activity (ARENA), Allied Health and Human Performance, University of South Australia, Adelaide, SA, Australia

^2^Australian Centre for Precision Health, Allied Health and Human Performance, University of South Australia, Adelaide, SA, Australia

**Corresponding author**

Lewis Ingram

Email: [Lewis.Ingram@unisa.edu.au](mailto:Lewis.Ingram@unisa.edu.au)

**Table S6** GRADE certainty of evidence assessments

| **Certainty assessment** | | | | | | | **№ of patients** | | **Effect** | | **Certainty** | **Importance** |
| --- | --- | --- | --- | --- | --- | --- | --- | --- | --- | --- | --- | --- |
| **№ of studies** | **Study design** | **Risk of bias** | **Inconsistency** | **Indirectness** | **Imprecision** | **Other considerations** | **Stretching** | **Control** | **Relative (95% CI)** | **Absolute (95% CI)** |  |  |
| **Acute SS on maximum tolerable PRT** | | | | | | | | | | | | |
| 10 | randomised trials | serious^a^ | not serious | not serious | serious^d^ | no publication bias suspected^h^ | 176 | 138 | - | SMD **0.25**  (-0.01 to 0.51) | ⨁⨁◯◯ Low | IMPORTANT |
| **Acute SS on overall stiffness** | | | | | | | | | | | | |
| 31 | randomised trials | serious^a^ | serious^b^ | not serious | not serious | publication bias strongly suspected^i^ | 896 | 762 | - | SMD **0.42**  (0.21 to 0.63) | ⨁◯◯◯ Very Low | IMPORTANT |

**Acute SS on fascicle length**

| 7 | randomised trials | serious^a^ | not serious | not serious | serious^e^ | no publication bias suspected^j^ | 117 | 109 | - | SMD **0.11**  (-0.26 to 0.47) | ⨁⨁◯◯ Low | IMPORTANT |
| --- | --- | --- | --- | --- | --- | --- | --- | --- | --- | --- | --- | --- |

**Chronic SS on maximum tolerable PRT**

| 17 | randomised trials | serious^a^ | serious^c^ | not serious | serious^f^ | publication bias strongly suspected^k^ | 267 | 231 | - | SMD **0.74**  (0.38 to 1.10) | ⨁◯◯◯ Very low | IMPORTANT |
| --- | --- | --- | --- | --- | --- | --- | --- | --- | --- | --- | --- | --- |

**Chronic SS on overall stiffness**

| 19 | randomised trials | serious^a^ | not serious | not serious | not serious | publication bias strongly suspected^l^ | 561 | 472 | - | SMD **0.37**  (0.18 to 0.56) | ⨁⨁◯◯ Low | IMPORTANT |
| --- | --- | --- | --- | --- | --- | --- | --- | --- | --- | --- | --- | --- |

**Chronic SS on fascicle length**

| 11 | randomised trials | serious^a^ | not serious | not serious | serious^g^ | no publication bias suspected^m^ | 171 | 142 | - | SMD **0.07**  (-0.25 to 0.26) | ⨁⨁◯◯ Low | IMPORTANT |
| --- | --- | --- | --- | --- | --- | --- | --- | --- | --- | --- | --- | --- |

**CI:** confidence interval; **SMD:** standardised mean difference

#### Explanations

a. >25% of participants were from trials with a PEDro score <5 out of 7.

b. Substantial heterogeneity was present in the primary analysis (*I*^2^ = 57.9%).

c. Substantial heterogeneity was present in the primary analysis (*I*^2^ = 65.4%).

d. n = 314 participants per outcome analysed

e. n = 226 participants per outcome analysed.

f. n = 498 participants per outcome analysed.

g. n = 313 participants per outcome analysed.

h. Egger’s test does not indicate publication bias (intercept = 0.76, *p* = 0.32).

i. Egger’s test indicated publication bias (intercept = 1.0, *p* = 0.007), and removal of five outliers from the primary analysis resulted in a reduction in the effect size estimate from *g* = 0.42 (small) to *g* = 0.33 (small).

j. Egger’s test does not indicate publication bias (intercept = 1.3, *p =* 0.300).

k. Egger’s test indicated publication bias (intercept = 2.2, *p* = 0.008), and removal of one outlier from the primary analysis resulted in an increase in the effect size estimate from *g* = 0.74 (moderate) to *g* = 0.82 (large).

l. Egger’s test indicated publication bias (intercept = 1.0, *p* = 0.010), and removal of one outlier from the primary analysis resulted in no change in the effect size estimate from *g* = 0.37 (small) to *g* = 0.37 (small).

m. Egger’s test does not indicate publication bias (intercept = 0.1, *p =* 0.74).
