# Supplementary material for: Mechanisms Underlying Range of Motion Improvements Following Acute and Chronic Static Stretching: A Systematic Review, Meta-analysis and Multivariate Meta-regression
Source: Sports Med. 2025 Apr 3;55(6):1449–66. doi: 10.1007/s40279-025-02204-7 (PMC12152101; doi:10.1007/s40279-025-02204-7)
Supplement: Supplementary file 2 — Supplementary file2 (DOCX 60 KB) [file 40279_2025_2204_MOESM2_ESM.docx]

**Title:** Mechanisms Underlying Range of Motion Improvements Following Acute and Chronic Static Stretching: A Systematic Review, Meta-Analysis, and Multivariate Meta-Regression

**Journal Name:** Sports Medicine

**Authors:** Lewis Ingram^1^, Grant Tomkinson^1^, Noah D’Unienville^1^, Bethany Gower^1^, Sam Gleadhill^1^, Terry Boyle^2^, and Hunter Bennett^1^

**Affiliations:**

^1^Alliance for Research in Exercise, Nutrition and Activity (ARENA), Allied Health and Human Performance, University of South Australia, Adelaide, SA, Australia

^2^Australian Centre for Precision Health, Allied Health and Human Performance, University of South Australia, Adelaide, SA, Australia

**Corresponding author**

Lewis Ingram

Email: Lewis.Ingram@unisa.edu.au

**Table S1** Search strategy for CINAHL Complete, Cochrane CENTRAL, Embase, Emcare, MEDLINE, Scopus, and SPORTSDiscus

**Search histories: static stretching AND flexibility AND controlled studies**

**All searches run 15 March 2023**

**MEDLINE (Ovid)**

**Link:**
[Click to run search](https://protect-au.mimecast.com/s/Vx5FCnx1rNSLx50pc9kvjN?domain=access.ovid.com)
The above Jumpstart will only work for users who have access to this specific database.

Database(s): **Ovid MEDLINE(R) ALL**1946 to March 13, 2023
Search Strategy:

| **#** | **Searches** | **Results** |
| --- | --- | --- |
| 1 | Muscle Stretching Exercises/ | 2014 |
| 2 | (stretch* adj3 (muscle* or isometric or static or passive or active or relaxed)).ti,ab,kf. | 6483 |
| 3 | (stretch* adj5 (hold* or held)).ti,ab,kf. | 349 |
| 4 | flexibility training.ti,ab,kf. | 262 |
| 5 | ((back or shoulder* or hamstring* or calf or quad* or glute* or hip* or thigh* or adductor* or iliotibial band) adj3 stretch*).ti,ab,kf. | 1137 |
| 6 | or/1-5 | 8648 |
| 7 | "Range of Motion, Articular"/ | 59034 |
| 8 | exp Joints/ and Movement/ | 10113 |
| 9 | Pliability/ | 4614 |
| 10 | exp Muscles/ and (length or fascicle angle or pennation angle or architecture).ti,ab,kf. | 26861 |
| 11 | Muscle Rigidity/ | 2265 |
| 12 | (flexib* or mobil* or ROM or range of motion or stiffness or rigidity or elongation).ti,ab,kf. | 817646 |
| 13 | (plantarflexion or dorsiflexion or flexion or extension or extensibility).ti,ab,kf. | 237631 |
| 14 | (musc* adj4 (length or fascicle angle or pennation angle or architecture)).ti,ab,kf. | 7812 |
| 15 | (stretch tolerance or passive torque).ti,ab,kf. | 298 |
| 16 | or/7-15 | 1077081 |
| 17 | randomized controlled trial/ | 588668 |
| 18 | controlled clinical trial/ | 95216 |
| 19 | Controlled Before-After Studies/ | 717 |
| 20 | (randomized or randomised or randomly).ab. | 1047198 |
| 21 | ((control or controlled) and (group* or study)).ab. | 2083628 |
| 22 | trial.ti,ab. | 741870 |
| 23 | groups.ab. | 2489607 |
| 24 | or/17-23 | 4681170 |
| 25 | exp animals/ not humans.sh. | 5102048 |
| 26 | 24 not 25 | 3954958 |
| 27 | 6 and 16 and 26 | 1650 |

**Embase (Ovid)**

**Link:**
[Click to run search](https://protect-au.mimecast.com/s/0GbiCvl1o9Hg4DDotQFKxz?domain=access.ovid.com)
The above Jumpstart will only work for users who have access to this specific database.

Database(s): **Embase Classic+Embase**1947 to 2023 March 13
Search Strategy:

| **#** | **Searches** | **Results** |
| --- | --- | --- |
| 1 | stretching/ | 7368 |
| 2 | muscle stretching/ | 6028 |
| 3 | stretching exercise/ | 4604 |
| 4 | (stretch* adj3 (muscle* or isometric or static or passive or active or relaxed)).ti,ab,kf. | 8608 |
| 5 | (stretch* adj5 (hold* or held)).ti,ab,kf. | 412 |
| 6 | flexibility training.ti,ab,kf. | 333 |
| 7 | ((back or shoulder* or hamstring* or calf or quad* or glute* or hip* or thigh* or adductor* or iliotibial band) adj3 stretch*).ti,ab,kf. | 1443 |
| 8 | or/1-7 | 23474 |
| 9 | "range of motion"/ | 60825 |
| 10 | exp joint/ and "movement (physiology)"/ | 5147 |
| 11 | pliability/ | 3337 |
| 12 | exp skeletal muscle/ and (length or fascicle angle or pennation angle or architecture).ti,ab,kf. | 22451 |
| 13 | joint mobility/ | 20175 |
| 14 | muscle length/ | 4250 |
| 15 | muscle rigidity/ | 11020 |
| 16 | rigidity/ | 58335 |
| 17 | ankle plantarflexion angle/ | 99 |
| 18 | ankle dorsiflexion angle/ | 141 |
| 19 | exp elbow angle/ | 79 |
| 20 | elbow flexion/ | 5361 |
| 21 | (flexib* or mobil* or ROM or range of motion or stiffness or rigidity or elongation).ti,ab,kf. | 1045332 |
| 22 | (plantarflexion or dorsiflexion or flexion or extension or extensibility).ti,ab,kf. | 319667 |
| 23 | (musc* adj4 (length or fascicle angle or pennation angle or architecture)).ti,ab,kf. | 9986 |
| 24 | (stretch tolerance or passive torque).ti,ab,kf. | 318 |
| 25 | or/9-24 | 1391594 |
| 26 | exp controlled study/ | 10022832 |
| 27 | (randomized or randomised or randomly).ab. | 1507565 |
| 28 | ((control or controlled) and (group* or study)).ab. | 2984924 |
| 29 | trial.ti,ab. | 1118344 |
| 30 | groups.ab. | 3621056 |
| 31 | or/26-30 | 13352333 |
| 32 | (rat or rats or mouse or mice or swine or porcine or murine or sheep or lambs or pigs or piglets or rabbit or rabbits or cat or cats or dog or dogs or cattle or bovine or monkey or monkeys or trout or marmoset or marmosets).ti. and animal experiment/ | 1217376 |
| 33 | Animal experiment/ not (human experiment/ or human/) | 2560259 |
| 34 | or/32-33 | 2627639 |
| 35 | 31 not 34 | 11560617 |
| 36 | 8 and 25 and 35 | 4234 |

**Emcare (Ovid)**

**Link:**
[Click to run search](https://protect-au.mimecast.com/s/P7RcC0YKVXSW4pp7cwQKMO?domain=access.ovid.com)
The above Jumpstart will only work for users who have access to this specific database.

Database(s): **Ovid Emcare**1995 to 2023 Week 09
Search Strategy:

| **#** | **Searches** | **Results** |
| --- | --- | --- |
| 1 | stretching/ | 3026 |
| 2 | muscle stretching/ | 1527 |
| 3 | stretching exercise/ | 1344 |
| 4 | (stretch* adj3 (muscle* or isometric or static or passive or active or relaxed)).ti,ab,kf. | 2572 |
| 5 | (stretch* adj5 (hold* or held)).ti,ab,kf. | 102 |
| 6 | flexibility training.ti,ab,kf. | 216 |
| 7 | ((back or shoulder* or hamstring* or calf or quad* or glute* or hip* or thigh* or adductor* or iliotibial band) adj3 stretch*).ti,ab,kf. | 765 |
| 8 | or/1-7 | 7114 |
| 9 | "range of motion"/ | 25511 |
| 10 | exp joint/ and "movement (physiology)"/ | 441 |
| 11 | pliability/ | 296 |
| 12 | exp skeletal muscle/ and (length or fascicle angle or pennation angle or architecture).ti,ab,kf. | 3755 |
| 13 | joint mobility/ | 8127 |
| 14 | muscle length/ | 1127 |
| 15 | muscle rigidity/ | 2100 |
| 16 | rigidity/ | 14108 |
| 17 | ankle plantarflexion angle/ | 3 |
| 18 | ankle dorsiflexion angle/ | 10 |
| 19 | exp elbow angle/ | 7 |
| 20 | elbow flexion/ | 1880 |
| 21 | (flexib* or mobil* or ROM or range of motion or stiffness or rigidity or elongation).ti,ab,kf. | 225560 |
| 22 | (plantarflexion or dorsiflexion or flexion or extension or extensibility).ti,ab,kf. | 82215 |
| 23 | (musc* adj4 (length or fascicle angle or pennation angle or architecture)).ti,ab,kf. | 2327 |
| 24 | (stretch tolerance or passive torque).ti,ab,kf. | 192 |
| 25 | or/9-24 | 306748 |
| 26 | exp controlled study/ | 1450343 |
| 27 | (randomized or randomised or randomly).ab. | 453398 |
| 28 | ((control or controlled) and (group* or study)).ab. | 686554 |
| 29 | trial.ti,ab. | 309060 |
| 30 | groups.ab. | 826293 |
| 31 | or/26-30 | 2385425 |
| 32 | (rat or rats or mouse or mice or swine or porcine or murine or sheep or lambs or pigs or piglets or rabbit or rabbits or cat or cats or dog or dogs or cattle or bovine or monkey or monkeys or trout or marmoset or marmosets).ti. and animal experiment/ | 95016 |
| 33 | Animal experiment/ not (human experiment/ or human/) | 184352 |
| 34 | or/32-33 | 192226 |
| 35 | 31 not 34 | 2222239 |
| 36 | 8 and 25 and 35 | 1835 |

**SPORTDiscus with Full Text (EBSCOhost)**

| **S1** | ( (DE ("STRETCH (Physiology)" OR "STATIC stretching (Physiology)")) ) OR ( (TI ((stretch* n3 (muscle* or isometric or static or passive or active or relaxed)) OR (stretch* n5 (hold* or held)) OR "flexibility training" OR ((back or shoulder* or hamstring* or calf or quad* or glute* or hip* or thigh* or adductor* or "iliotibial band") n3 stretch*))) OR (AB ((stretch* n3 (muscle* or isometric or static or passive or active or relaxed)) OR (stretch* n5 (hold* or held)) OR "flexibility training" OR ((back or shoulder* or hamstring* or calf or quad* or glute* or hip* or thigh* or adductor* or "iliotibial band") n3 stretch*))) OR (KW ((stretch* n3 (muscle* or isometric or static or passive or active or relaxed)) OR (stretch* n5 (hold* or held)) OR "flexibility training" OR ((back or shoulder* or hamstring* or calf or quad* or glute* or hip* or thigh* or adductor* or "iliotibial band") n3 stretch*))) ) | (7,323) |
| --- | --- | --- |
| **S2** | ( (DE ("RANGE of motion of joints" OR "JOINT stiffness" OR "MUSCLE rigidity" OR "PLANTARFLEXION" OR "DORSIFLEXION" OR "HIP flexion" OR "WRIST extension" OR "WRIST flexion")) ) OR ( (DE ("JOINTS (Anatomy)" OR "ACROMIOCLAVICULAR joint" OR "ANKLE joint" OR "ARTICULAR cartilage" OR "ARTICULAR ligaments" OR "ATLANTO-axial joint" OR "ATLANTO-occipital joint" OR "CRANIOVERTEBRAL junction" OR "ELBOW joint" OR "FINGER joint" OR "GLENOHUMERAL joint" OR "HIP joint" OR "KNEE joint" OR "MANDIBULAR joint" OR "SACROILIAC joint" OR "SHOULDER joint" OR "STIFLE joint" OR "TARSAL joint" OR "TOE joint" OR "WRIST joint" OR "ZYGAPOPHYSEAL joint") AND (DE ("BODY movement")))) ) OR ( (DE ("SKELETAL muscle" OR "ABDOMINAL muscles" OR "BACK muscles" OR "CALF muscles" OR "DELTOID muscles" OR "GLUTEAL muscles" OR "LATISSIMUS dorsi (Muscles)" OR "LEG muscles" OR "NECK muscles" OR "PECTORALIS muscle" OR "RESPIRATORY muscles" OR "CALF muscles" OR "HAMSTRING muscle" OR "PERONEUS brevis" OR "PERONEUS longus" OR "QUADRICEPS muscle" OR "THIGH muscles" OR "TIBIALIS anterior" OR "TIBIALIS posterior" OR "VASTUS lateralis" OR "SCALENE muscles" OR "SPLENIUS muscles" OR "STERNOCLEIDOMASTOID muscle" OR "GLUTEUS medius" OR "GLUTEUS minimus" OR "PIRIFORMIS muscle" OR "SOLEUS muscle" OR "SPLENIUS muscles" OR "TRAPEZIUS muscle")) AND ((TI (length or "fascicle angle" or "pennation angle" or architecture)) OR (AB (length or "fascicle angle" or "pennation angle" or architecture)) OR (KW (length or "fascicle angle" or "pennation angle" or architecture))) ) OR ( (TI ((flexib* or mobil* or ROM or "range of motion" or stiffness or rigidity OR elongation OR plantarflexion or dorsiflexion or flexion or extension or extensibility OR (musc* n4 (length or "fascicle angle" or "pennation angle" or architecture)) OR "stretch tolerance" or "passive torque"))) OR (AB ((flexib* or mobil* or ROM or "range of motion" or stiffness or rigidity OR elongation OR plantarflexion or dorsiflexion or flexion or extension or extensibility OR (musc* n4 (length or "fascicle angle" or "pennation angle" or architecture)) OR "stretch tolerance" or "passive torque")) OR (KW ((flexib* or mobil* or ROM or "range of motion" or stiffness or rigidity OR elongation OR plantarflexion or dorsiflexion or flexion or extension or extensibility OR (musc* n4 (length or "fascicle angle" or "pennation angle" or architecture)) OR "stretch tolerance" or "passive torque"))) ) | (86,520) |
| **S3** | ( (DE ("RANDOMIZED controlled trials" OR "CLINICAL trials")) ) OR ( (TI (trial)) OR (AB (trial)) ) OR ( (AB (randomized or randomised or randomly OR ((control or controlled) and (group* or study)) OR groups)) ) | (269,583) |
| **S4** | S1 AND S2 AND S3 | (1,642) |

**CINAHL Complete (EBSCOhost)**

| **S1** | ( (MH ("Stretching")) ) OR ( (TI ((stretch* n3 (muscle* or isometric or static or passive or active or relaxed)) OR (stretch* n5 (hold* or held)) OR "flexibility training" OR ((back or shoulder* or hamstring* or calf or quad* or glute* or hip* or thigh* or adductor* or "iliotibial band") n3 stretch*))) OR (AB ((stretch* n3 (muscle* or isometric or static or passive or active or relaxed)) OR (stretch* n5 (hold* or held)) OR "flexibility training" OR ((back or shoulder* or hamstring* or calf or quad* or glute* or hip* or thigh* or adductor* or "iliotibial band") n3 stretch*))) ) | (7,159) |
| --- | --- | --- |
| **S2** | ( (MH ("Range of Motion" OR ("Joints+" AND "Movement") OR "Pliability" OR "Extension" OR "Flexion+")) ) OR ( (MH ("Muscle, Skeletal+")) AND ((TI (length or "fascicle angle" or "pennation angle" or architecture)) OR (AB (length or "fascicle angle" or "pennation angle" or architecture))) ) OR ( (TI ((flexib* or mobil* or ROM or "range of motion" or stiffness or rigidity OR elongation OR plantarflexion or dorsiflexion or flexion or extension or extensibility OR (musc* n4 (length or "fascicle angle" or "pennation angle" or architecture)) OR "stretch tolerance" or "passive torque"))) OR (AB ((flexib* or mobil* or ROM or "range of motion" or stiffness or rigidity OR elongation OR plantarflexion or dorsiflexion or flexion or extension or extensibility OR (musc* n4 (length or "fascicle angle" or "pennation angle" or architecture)) OR "stretch tolerance" or "passive torque"))) ) | (214,460) |
| **S3** | ( (MH ("Clinical Trials+" OR "Controlled Before-After Studies")) ) OR ( (TI (trial)) OR (AB (trial)) ) OR ( (AB (randomized or randomised or randomly OR ((control or controlled) and (group* or study)) OR groups)) ) | (1,513,408) |
| **S4** | (MH ("Animals+" OR "Animal Studies")) OR (TI ("Animal Model*")) | (248,095) |
| **S5** | (MH (Human)) | (2,664,471) |
| **S6** | S4 NOT S5 | (214,113) |
| **S7** | S1 AND S2 AND S3 | (1,490) |
| **S8** | S7 NOT S6 | (1,462) |

**Scopus**

(( TITLE-ABS-KEY ( ( stretch* W/3 ( muscle* OR isometric OR static OR passive OR active OR relaxed OR back OR shoulder* OR hamstring* OR calf OR quad* OR glute* OR hip* OR thigh* OR adductor* OR "iliotibial band" ) ) OR ( stretch* W/5 ( hold* OR held ) ) OR "flexibility training" ) ) AND ( TITLE-ABS-KEY ( flexib* OR mobil* OR rom OR "range of motion" OR stiffness OR rigidity OR elongation OR plantarflexion OR dorsiflexion OR flexion OR extension OR extensibility OR ( musc* W/4 ( length OR "fascicle angle" OR "pennation angle" OR architecture ) ) OR "stretch tolerance" OR "passive torque" ) ) AND ( TITLE-ABS ( trial ) OR ABS ( randomized OR randomised OR randomly OR ( ( control OR controlled ) AND ( group* OR study ) ) OR groups ) )) AND NOT (TITLE-ABS-KEY((animal* OR avian OR baboon* OR bird* OR bovine OR canine OR cat OR cats OR cattle OR chick* OR cow OR cows OR dog OR dogs OR feline* OR fish* OR frog* OR geese OR goose OR lamb OR lambs OR macaque* OR marmoset OR marmosets OR mice OR monkey OR monkeys OR mouse OR murine OR nonhuman OR "non human" OR ovine OR piglet OR piglets OR porcine OR primate* OR rabbit OR rabbits OR rat OR rats OR rodent* OR sheep OR swine OR trout) AND NOT (human OR humans OR patient OR patients OR woman OR women OR man OR men OR athlete* OR person OR people)))

2,675 document results

**Cochrane CENTRAL**

ID Search Hits

#1 MeSH descriptor: [Muscle Stretching Exercises] this term only 776

#2 (stretch* near/3 (muscle* or isometric or static or passive or active or relaxed)):ti,ab,kw 2994

#3 (stretch* near/5 (hold* or held)):ti,ab,kw 195

#4 ("flexibility training"):ti,ab,kw 228

#5 ((back or shoulder* or hamstring* or calf or quad* or glute* or hip* or thigh* or adductor* or "iliotibial band") near/3 stretch*):ti,ab,kw 985

#6 {OR #1-#5} 3695

#7 MeSH descriptor: [Range of Motion, Articular] this term only 5889

#8 [mh "Joints"] AND [mh ^"Movement"] 399

#9 MeSH descriptor: [Pliability] this term only 249

#10 [mh "Muscles"] AND (length or "fascicle angle" or "pennation angle" or architecture):ti,ab,kw 796

#11 MeSH descriptor: [Muscle Rigidity] this term only 76

#12 (flexib* or mobil* or ROM or "range of motion" or stiffness or rigidity OR elongation):ti,ab,kw 79397

#13 (plantarflexion or dorsiflexion or flexion or extension or extensibility):ti,ab,kw 31473

#14 (musc* near/4 (length or "fascicle angle" or "pennation angle" or architecture)):ti,ab,kw 780

#15 ("stretch tolerance" or "passive torque"):ti,ab,kw 100

#16 {OR #7-#15} 103915

#17 #6 AND #16 2468

**Search histories: static stretching AND flexibility AND controlled studies**

**All searches run 06 June 2024**

**MEDLINE (Ovid)**

**Link:**
[Click to run search](https://url.au.m.mimecastprotect.com/s/jfhUCE8w96t360Q7wiNVG9x?domain=access.ovid.com)
The above Jumpstart will only work for users who have access to this specific database.

**Database:**
Ovid MEDLINE(R) ALL <1946 to June 04, 2024>

| **#** | **Query** | **Results from 6 Jun 2024** |
| --- | --- | --- |
| 1 | Muscle Stretching Exercises/ | 2,098 |
| 2 | (stretch* adj3 (muscle* or isometric or static or passive or active or relaxed)).ti,ab,kf. | 6,782 |
| 3 | (stretch* adj5 (hold* or held)).ti,ab,kf. | 360 |
| 4 | flexibility training.ti,ab,kf. | 289 |
| 5 | ((back or shoulder* or hamstring* or calf or quad* or glute* or hip* or thigh* or adductor* or iliotibial band) adj3 stretch*).ti,ab,kf. | 1,200 |
| 6 | or/1-5 | 9,045 |
| 7 | "Range of Motion, Articular"/ | 61,191 |
| 8 | exp Joints/ and Movement/ | 10,261 |
| 9 | Pliability/ | 4,630 |
| 10 | exp Muscles/ and (length or fascicle angle or pennation angle or architecture).ti,ab,kf. | 27,833 |
| 11 | Muscle Rigidity/ | 2,276 |
| 12 | (flexib* or mobil* or ROM or range of motion or stiffness or rigidity or elongation).ti,ab,kf. | 889,883 |
| 13 | (plantarflexion or dorsiflexion or flexion or extension or extensibility).ti,ab,kf. | 253,928 |
| 14 | (musc* adj4 (length or fascicle angle or pennation angle or architecture)).ti,ab,kf. | 8,270 |
| 15 | (stretch tolerance or passive torque).ti,ab,kf. | 321 |
| 16 | or/7-15 | 1,164,190 |
| 17 | randomized controlled trial/ | 614,484 |
| 18 | controlled clinical trial/ | 95,544 |
| 19 | Controlled Before-After Studies/ | 758 |
| 20 | (randomized or randomised or randomly).ab. | 1,132,176 |
| 21 | ((control or controlled) and (group* or study)).ab. | 2,250,115 |
| 22 | trial.ti,ab. | 810,698 |
| 23 | groups.ab. | 2,687,527 |
| 24 | or/17-23 | 5,044,050 |
| 25 | exp animals/ not humans.sh. | 5,228,387 |
| 26 | 24 not 25 | 4,289,116 |
| 27 | 6 and 16 and 26 | 1,774 |
| 28 | limit 27 to dt=20230314-20240606 | 128 |

**Embase (Ovid)**

**Link:**
[Click to run search](https://url.au.m.mimecastprotect.com/s/ebyCC3QN2KTpR96oLSgyCgF?domain=access.ovid.com)
The above Jumpstart will only work for users who have access to this specific database.


**Database:**
Embase Classic+Embase <1947 to 2024 June 04>

| **#** | **Query** | **Results from 6 Jun 2024** |
| --- | --- | --- |
| 1 | stretching/ | 8,249 |
| 2 | muscle stretching/ | 6,213 |
| 3 | stretching exercise/ | 5,325 |
| 4 | (stretch* adj3 (muscle* or isometric or static or passive or active or relaxed)).ti,ab,kf. | 8,945 |
| 5 | (stretch* adj5 (hold* or held)).ti,ab,kf. | 431 |
| 6 | flexibility training.ti,ab,kf. | 365 |
| 7 | ((back or shoulder* or hamstring* or calf or quad* or glute* or hip* or thigh* or adductor* or iliotibial band) adj3 stretch*).ti,ab,kf. | 1,527 |
| 8 | or/1-7 | 25,430 |
| 9 | "range of motion"/ | 69,168 |
| 10 | exp joint/ and "movement (physiology)"/ | 2,351 |
| 11 | pliability/ | 3,465 |
| 12 | exp skeletal muscle/ and (length or fascicle angle or pennation angle or architecture).ti,ab,kf. | 24,057 |
| 13 | joint mobility/ | 21,747 |
| 14 | muscle length/ | 4,391 |
| 15 | muscle rigidity/ | 11,759 |
| 16 | rigidity/ | 60,193 |
| 17 | ankle plantarflexion angle/ | 299 |
| 18 | ankle dorsiflexion angle/ | 454 |
| 19 | exp elbow angle/ | 170 |
| 20 | elbow flexion/ | 5,809 |
| 21 | (flexib* or mobil* or ROM or range of motion or stiffness or rigidity or elongation).ti,ab,kf. | 1,116,333 |
| 22 | (plantarflexion or dorsiflexion or flexion or extension or extensibility).ti,ab,kf. | 338,054 |
| 23 | (musc* adj4 (length or fascicle angle or pennation angle or architecture)).ti,ab,kf. | 10,476 |
| 24 | (stretch tolerance or passive torque).ti,ab,kf. | 340 |
| 25 | or/9-24 | 1,483,025 |
| 26 | exp controlled study/ | 10,622,758 |
| 27 | (randomized or randomised or randomly).ab. | 1,603,359 |
| 28 | ((control or controlled) and (group* or study)).ab. | 3,173,669 |
| 29 | trial.ti,ab. | 1,196,916 |
| 30 | groups.ab. | 3,860,874 |
| 31 | or/26-30 | 14,151,650 |
| 32 | (rat or rats or mouse or mice or swine or porcine or murine or sheep or lambs or pigs or piglets or rabbit or rabbits or cat or cats or dog or dogs or cattle or bovine or monkey or monkeys or trout or marmoset or marmosets).ti. and animal experiment/ | 1,254,614 |
| 33 | Animal experiment/ not (human experiment/ or human/) | 2,641,554 |
| 34 | or/32-33 | 2,715,736 |
| 35 | 31 not 34 | 12,283,672 |
| 36 | 8 and 25 and 35 | 4,723 |
| 37 | limit 36 to dc=20230314-20240606 | 549 |

**Emcare (Ovid)**

**Link:**
[Click to run search](https://url.au.m.mimecastprotect.com/s/49vLCk81ZOtOqX9gPu2D8bF?domain=access.ovid.com)
The above Jumpstart will only work for users who have access to this specific database.


**Database:**
Ovid Emcare <1995 to 2024 Week 22>

| **#** | **Query** | **Results from 6 Jun 2024** |
| --- | --- | --- |
| 1 | stretching/ | 3,127 |
| 2 | muscle stretching/ | 1,528 |
| 3 | stretching exercise/ | 1,417 |
| 4 | (stretch* adj3 (muscle* or isometric or static or passive or active or relaxed)).ti,ab,kf. | 2,734 |
| 5 | (stretch* adj5 (hold* or held)).ti,ab,kf. | 105 |
| 6 | flexibility training.ti,ab,kf. | 240 |
| 7 | ((back or shoulder* or hamstring* or calf or quad* or glute* or hip* or thigh* or adductor* or iliotibial band) adj3 stretch*).ti,ab,kf. | 825 |
| 8 | or/1-7 | 7,448 |
| 9 | "range of motion"/ | 26,302 |
| 10 | exp joint/ and "movement (physiology)"/ | 441 |
| 11 | pliability/ | 302 |
| 12 | exp skeletal muscle/ and (length or fascicle angle or pennation angle or architecture).ti,ab,kf. | 3,932 |
| 13 | joint mobility/ | 8,212 |
| 14 | muscle length/ | 1,155 |
| 15 | muscle rigidity/ | 2,128 |
| 16 | rigidity/ | 14,503 |
| 17 | ankle plantarflexion angle/ | 4 |
| 18 | ankle dorsiflexion angle/ | 20 |
| 19 | exp elbow angle/ | 17 |
| 20 | elbow flexion/ | 1,925 |
| 21 | (flexib* or mobil* or ROM or range of motion or stiffness or rigidity or elongation).ti,ab,kf. | 249,305 |
| 22 | (plantarflexion or dorsiflexion or flexion or extension or extensibility).ti,ab,kf. | 89,489 |
| 23 | (musc* adj4 (length or fascicle angle or pennation angle or architecture)).ti,ab,kf. | 2,533 |
| 24 | (stretch tolerance or passive torque).ti,ab,kf. | 204 |
| 25 | or/9-24 | 336,482 |
| 26 | exp controlled study/ | 1,537,224 |
| 27 | (randomized or randomised or randomly).ab. | 498,101 |
| 28 | ((control or controlled) and (group* or study)).ab. | 754,079 |
| 29 | trial.ti,ab. | 343,946 |
| 30 | groups.ab. | 912,362 |
| 31 | or/26-30 | 2,592,410 |
| 32 | (rat or rats or mouse or mice or swine or porcine or murine or sheep or lambs or pigs or piglets or rabbit or rabbits or cat or cats or dog or dogs or cattle or bovine or monkey or monkeys or trout or marmoset or marmosets).ti. and animal experiment/ | 96,611 |
| 33 | Animal experiment/ not (human experiment/ or human/) | 186,675 |
| 34 | or/32-33 | 194,756 |
| 35 | 31 not 34 | 2,426,998 |
| 36 | 8 and 25 and 35 | 1,956 |
| 37 | limit 36 to dc=20230314-20240606 | 119 |

**SPORTDiscus with Full Text (EBSCOhost)**

| **S1** | ( (DE ("STRETCH (Physiology)" OR "STATIC stretching (Physiology)")) ) OR ( (TI ((stretch* n3 (muscle* or isometric or static or passive or active or relaxed)) OR (stretch* n5 (hold* or held)) OR "flexibility training" OR ((back or shoulder* or hamstring* or calf or quad* or glute* or hip* or thigh* or adductor* or "iliotibial band") n3 stretch*))) OR (AB ((stretch* n3 (muscle* or isometric or static or passive or active or relaxed)) OR (stretch* n5 (hold* or held)) OR "flexibility training" OR ((back or shoulder* or hamstring* or calf or quad* or glute* or hip* or thigh* or adductor* or "iliotibial band") n3 stretch*))) OR (KW ((stretch* n3 (muscle* or isometric or static or passive or active or relaxed)) OR (stretch* n5 (hold* or held)) OR "flexibility training" OR ((back or shoulder* or hamstring* or calf or quad* or glute* or hip* or thigh* or adductor* or "iliotibial band") n3 stretch*))) ) | (7,622) |
| --- | --- | --- |
| **S2** | ( (DE ("RANGE of motion of joints" OR "JOINT stiffness" OR "MUSCLE rigidity" OR "PLANTARFLEXION" OR "DORSIFLEXION" OR "HIP flexion" OR "WRIST extension" OR "WRIST flexion")) ) OR ( (DE ("JOINTS (Anatomy)" OR "ACROMIOCLAVICULAR joint" OR "ANKLE joint" OR "ARTICULAR cartilage" OR "ARTICULAR ligaments" OR "ATLANTO-axial joint" OR "ATLANTO-occipital joint" OR "CRANIOVERTEBRAL junction" OR "ELBOW joint" OR "FINGER joint" OR "GLENOHUMERAL joint" OR "HIP joint" OR "KNEE joint" OR "MANDIBULAR joint" OR "SACROILIAC joint" OR "SHOULDER joint" OR "STIFLE joint" OR "TARSAL joint" OR "TOE joint" OR "WRIST joint" OR "ZYGAPOPHYSEAL joint") AND (DE ("BODY movement")))) ) OR ( (DE ("SKELETAL muscle" OR "ABDOMINAL muscles" OR "BACK muscles" OR "CALF muscles" OR "DELTOID muscles" OR "GLUTEAL muscles" OR "LATISSIMUS dorsi (Muscles)" OR "LEG muscles" OR "NECK muscles" OR "PECTORALIS muscle" OR "RESPIRATORY muscles" OR "CALF muscles" OR "HAMSTRING muscle" OR "PERONEUS brevis" OR "PERONEUS longus" OR "QUADRICEPS muscle" OR "THIGH muscles" OR "TIBIALIS anterior" OR "TIBIALIS posterior" OR "VASTUS lateralis" OR "SCALENE muscles" OR "SPLENIUS muscles" OR "STERNOCLEIDOMASTOID muscle" OR "GLUTEUS medius" OR "GLUTEUS minimus" OR "PIRIFORMIS muscle" OR "SOLEUS muscle" OR "SPLENIUS muscles" OR "TRAPEZIUS muscle")) AND ((TI (length or "fascicle angle" or "pennation angle" or architecture)) OR (AB (length or "fascicle angle" or "pennation angle" or architecture)) OR (KW (length or "fascicle angle" or "pennation angle" or architecture))) ) OR ( (TI ((flexib* or mobil* or ROM or "range of motion" or stiffness or rigidity OR elongation OR plantarflexion or dorsiflexion or flexion or extension or extensibility OR (musc* n4 (length or "fascicle angle" or "pennation angle" or architecture)) OR "stretch tolerance" or "passive torque"))) OR (AB ((flexib* or mobil* or ROM or "range of motion" or stiffness or rigidity OR elongation OR plantarflexion or dorsiflexion or flexion or extension or extensibility OR (musc* n4 (length or "fascicle angle" or "pennation angle" or architecture)) OR "stretch tolerance" or "passive torque")) OR (KW ((flexib* or mobil* or ROM or "range of motion" or stiffness or rigidity OR elongation OR plantarflexion or dorsiflexion or flexion or extension or extensibility OR (musc* n4 (length or "fascicle angle" or "pennation angle" or architecture)) OR "stretch tolerance" or "passive torque"))) ) | (91,738) |
| **S3** | ( (DE ("RANDOMIZED controlled trials" OR "CLINICAL trials")) ) OR ( (TI (trial)) OR (AB (trial)) ) OR ( (AB (randomized or randomised or randomly OR ((control or controlled) and (group* or study)) OR groups)) ) | (286,115) |
| **S4** | S1 AND S2 AND S3 | (1,780) |
| **S5** | ED 20230314-20240606 | (71,486) |
| **S6** | S4 AND S5 | (147) |

**CINAHL Complete (EBSCOhost)**

| **S1** | ( (MH ("Stretching")) ) OR ( (TI ((stretch* n3 (muscle* or isometric or static or passive or active or relaxed)) OR (stretch* n5 (hold* or held)) OR "flexibility training" OR ((back or shoulder* or hamstring* or calf or quad* or glute* or hip* or thigh* or adductor* or "iliotibial band") n3 stretch*))) OR (AB ((stretch* n3 (muscle* or isometric or static or passive or active or relaxed)) OR (stretch* n5 (hold* or held)) OR "flexibility training" OR ((back or shoulder* or hamstring* or calf or quad* or glute* or hip* or thigh* or adductor* or "iliotibial band") n3 stretch*))) ) | (6,956) |
| --- | --- | --- |
| **S2** | ( (MH ("Range of Motion" OR ("Joints+" AND "Movement") OR "Pliability" OR "Extension" OR "Flexion+")) ) OR ( (MH ("Muscle, Skeletal+")) AND ((TI (length or "fascicle angle" or "pennation angle" or architecture)) OR (AB (length or "fascicle angle" or "pennation angle" or architecture))) ) OR ( (TI ((flexib* or mobil* or ROM or "range of motion" or stiffness or rigidity OR elongation OR plantarflexion or dorsiflexion or flexion or extension or extensibility OR (musc* n4 (length or "fascicle angle" or "pennation angle" or architecture)) OR "stretch tolerance" or "passive torque"))) OR (AB ((flexib* or mobil* or ROM or "range of motion" or stiffness or rigidity OR elongation OR plantarflexion or dorsiflexion or flexion or extension or extensibility OR (musc* n4 (length or "fascicle angle" or "pennation angle" or architecture)) OR "stretch tolerance" or "passive torque"))) ) | (216,503) |
| **S3** | ( (MH ("Clinical Trials+" OR "Controlled Before-After Studies")) ) OR ( (TI (trial)) OR (AB (trial)) ) OR ( (AB (randomized or randomised or randomly OR ((control or controlled) and (group* or study)) OR groups)) ) | (1,551,295) |
| **S4** | (MH ("Animals+" OR "Animal Studies")) OR (TI ("Animal Model*")) | (249,664) |
| **S5** | (MH (Human)) | (2,802,064) |
| **S6** | S4 NOT S5 | (215,058) |
| **S7** | S1 AND S2 AND S3 | (1,551) |
| **S8** | S7 NOT S6 | (1,524) |
| **S9** | EM 20230314-20240606 | (307,657) |
| **S10** | S8 AND S9 | (102) |

**Scopus**

(((TITLE-ABS-KEY ( ( stretch* W/3 ( muscle* OR isometric OR static OR passive OR active OR relaxed OR back OR shoulder* OR hamstring* OR calf OR quad* OR glute* OR hip* OR thigh* OR adductor* OR "iliotibial band" ) ) OR ( stretch* W/5 ( hold* OR held ) ) OR "flexibility training" ) ) AND ( TITLE-ABS-KEY ( flexib* OR mobil* OR rom OR "range of motion" OR stiffness OR rigidity OR elongation OR plantarflexion OR dorsiflexion OR flexion OR extension OR extensibility OR ( musc* W/4 ( length OR "fascicle angle" OR "pennation angle" OR architecture ) ) OR "stretch tolerance" OR "passive torque" ) ) AND ( TITLE-ABS ( trial ) OR ABS ( randomized OR randomised OR randomly OR ( ( control OR controlled ) AND ( group* OR study ) ) OR groups ) )) AND NOT (TITLE-ABS-KEY((animal* OR avian OR baboon* OR bird* OR bovine OR canine OR cat OR cats OR cattle OR chick* OR cow OR cows OR dog OR dogs OR feline* OR fish* OR frog* OR geese OR goose OR lamb OR lambs OR macaque* OR marmoset OR marmosets OR mice OR monkey OR monkeys OR mouse OR murine OR nonhuman OR "non human" OR ovine OR piglet OR piglets OR porcine OR primate* OR rabbit OR rabbits OR rat OR rats OR rodent* OR sheep OR swine OR trout) AND NOT (human OR humans OR patient OR patients OR woman OR women OR man OR men OR athlete* OR person OR people)))) and ORIG-LOAD-DATE > 20230313

251 document results [however only 250 will export out - one record is being lost somewhere but can't determine what might be happening here]

**Cochrane CENTRAL**

Date Run: 06/06/2024 09:05:06

ID Search Hits

#1 MeSH descriptor: [Muscle Stretching Exercises] this term only 877

#2 (stretch* near/3 (muscle* or isometric or static or passive or active or relaxed)):ti,ab,kw 3462

#3 (stretch* near/5 (hold* or held)):ti,ab,kw 242

#4 ("flexibility training"):ti,ab,kw 261

#5 ((back or shoulder* or hamstring* or calf or quad* or glute* or hip* or thigh* or adductor* or "iliotibial band") near/3 stretch*):ti,ab,kw 1214

#6 {OR #1-#5} 4344

#7 MeSH descriptor: [Range of Motion, Articular] this term only 6735

#8 [mh "Joints"] AND [mh ^"Movement"] 451

#9 MeSH descriptor: [Pliability] this term only 261

#10 [mh "Muscles"] AND (length or "fascicle angle" or "pennation angle" or architecture):ti,ab,kw 945

#11 MeSH descriptor: [Muscle Rigidity] this term only 82

#12 (flexib* or mobil* or ROM or "range of motion" or stiffness or rigidity OR elongation):ti,ab,kw 91739

#13 (plantarflexion or dorsiflexion or flexion or extension or extensibility):ti,ab,kw 35615

#14 (musc* near/4 (length or "fascicle angle" or "pennation angle" or architecture)):ti,ab,kw 903

#15 ("stretch tolerance" or "passive torque"):ti,ab,kw 110

#16 {OR #7-#15} 119294

#17 #6 AND #16 with Cochrane Library publication date Between Mar 2023 and Jun 2024 425
