# Supplementary material for: Mechanisms Underlying Range of Motion Improvements Following Acute and Chronic Static Stretching: A Systematic Review, Meta-analysis and Multivariate Meta-regression
Source: Sports Med. 2025 Apr 3;55(6):1449–66. doi: 10.1007/s40279-025-02204-7 (PMC12152101; doi:10.1007/s40279-025-02204-7)
Supplement: Supplementary file 3 — Supplementary file3 (DOCX 46 KB) [file 40279_2025_2204_MOESM3_ESM.docx]

**Title:** Mechanisms Underlying Range of Motion Improvements Following Acute and Chronic Static Stretching: A Systematic Review, Meta-Analysis, and Multivariate Meta-Regression

**Journal Name:** Sports Medicine

**Authors:** Lewis Ingram^1^, Grant Tomkinson^1^, Noah D’Unienville^1^, Bethany Gower^1^, Sam Gleadhill^1^, Terry Boyle^2^, and Hunter Bennett^1^

**Affiliations:**

^1^Alliance for Research in Exercise, Nutrition and Activity (ARENA), Allied Health and Human Performance, University of South Australia, Adelaide, SA, Australia

^2^Australian Centre for Precision Health, Allied Health and Human Performance, University of South Australia, Adelaide, SA, Australia

**Corresponding author**

Lewis Ingram

Email: [Lewis.Ingram@unisa.edu.au](mailto:Lewis.Ingram@unisa.edu.au)

**Table S2** Characteristics of included studies

| **Study** | **Study design** | **Sample size (n)** | **Intervention group (n)** | **Control group (n)** | **Age** | **Participants’ health status** | **Participants’ activity level** | **Participants’ baseline flexibility** | **Main outcome** | **PEDro scale score** | **Adjusted PEDro scale score** |
| --- | --- | --- | --- | --- | --- | --- | --- | --- | --- | --- | --- |
| Akagi & Takahashi (2014) | RCT (contralateral extremity control group) | n = 19  Males (n = 19)  Females (n = 0) | Unilateral design |  | 23.7 ± 2.3 | Healthy | Sedentary (n = 6)  Recreationally active (n = 13) | Not reported | MTU stiffness;  Shear elastic modulus;  Passive dorsiflexion ROM | 5 | 5 |
| Andrade et al. (2020) | RCT (independent control group) | n = 60  Males (n = 31)  Females (n = 29) | n = 21  Males (n = 10)  Females (n = 11) | n = 18  Males (n = 9)  Females n = 9) | 20.5 ± 2  IG = 21 ± 2.4  CG = 21.1 ± 2 | Healthy | Unclear | Not reported | PRT at a given angle;  Shear elastic modulus;  Fascicle length;  Passive dorsiflexion ROM | 6 | 6 |
| Aquino et al. (2010) | RCT (independent control group) | n = 45  Males (n = 6)  Females (n = 39) | n = 15  Males (n = 2)  Females (n = 13) | n = 15  Males (n = 2)  Females (n = 13) | IG = 22.6 ± 1.8  CG = 22.3 ± 1.5 | Healthy | Unclear | Limited | Maximum tolerable PRT;  Passive knee extension test | 4 | 3 |
| Barbosa et al. (2018) | RCT (independent control group) | n = 45  Males (n = 45)  Females (n = 0) | n = 15 | n = 15 | IG = 23.1 ± 3.5  CG = 21.3 ± 2.8 | Healthy | Trained | Limited | Maximum tolerable PRT;  Active knee extension test | 5 | 4 |
| Ben & Harvey (2010) | RCT (independent *and* contralateral extremity control group) | n = 60  Males (n = 16)  Females (n = 44) | n = 30  Males (n = 9)  Females (n = 21) | n = 30  Males (n = 7)  Females (n = 7) | IG = 35 ± 12  CG = 39 ± 12 | Healthy | Recreationally active | Normal | Maximum tolerable PRT | 8 | 7 |
| Blazevich et al. (2014) | RCT (independent control group) | n = 22  Males (n = 22)  Females (n = 0) | n = 12 | n = 9 | 18.6 ± 0.9 | Healthy | Unclear | Not reported | MTU stiffness;  Muscle stiffness;  Tendon stiffness;  Fascicle length;  Passive dorsiflexion ROM | 5 | 5 |
| Cannavan et al. (2012) | CT (crossover study design) | n = 18  Males (n = 9)  Females (n = 9) | RCT (crossover study design) |  | Males = 20.4 ± 0.7  Females = 20 ± 2.4 | Healthy | Recreationally active | Not reported | PRT at a given angle;  Maximum tolerable PRT | 2 | 2 |
| Cini et al. (2024) | RCT (independent control group) | n = 30  Males (n = 6)  Females (n = 24) | IG (1) n = 10  Males (n = 1)  Females (n = 9)  IG (2) n = 10  Males (n = 2)  Females (n = 8) | n = 10  Males (n = 3)  Females (n = 7) | IG (1) = 22.6 ± 3  IG (2) = 24.9 ± 6.4  CG = 23.8 ± 3.7 | Healthy | Recreationally active | Not reported | PRT at a given angle;  Maximum tolerable PRT; MTU stiffness;  Muscle stiffness;  Tendon stiffness;  Passive dorsiflexion ROM | 7 | 6 |
| de Oliveira et al. (2018) | RCT (independent control group) | n = 31  Males (n = 11)  Females (n = 20) | n = 15  Males (n = 3)  Females (n = 12) | n = 16  Males (n = 8)  Females (n = 8) | IG = 21.4 ± 1.4  CG = 21 ± 1.9 | Healthy | Trained | Limited | MTU stiffness;  Passive knee extension test | 3 | 3 |
| e Lima et al.  (2015) | RCT (independent control group) | n = 24  Males (n = 24)  Females (n = 0) | n = 12 | n = 10 | 19.1 ± 1.4 | Healthy | Recreationally active | Not reported | Fascicle length;  Knee flexion passive ROM;  Passive knee extension test | 5 | 4 |
| Farrow et al. (2024) | RCT (crossover study design) | n = 30  Males (n = 0)  Females (n = 30) | Crossover design | n = 30  Young (n = 15)  Older (n = 15) | Young = 23.0 ± 3.9  Older = 72.6 ± 5.3 | Healthy | Recreationally active | Not reported | MTU stiffness | 4 | 4 |
| Folpp et al. (2006) | RCT (contralateral extremity control group) | n = 20  Males (n = 8)  Females (n = 12) | Unilateral design |  | 24 ± 6.5 | Healthy | Unclear | Limited | Maximum tolerable PRT;  Passive straight leg raise | 8 | 7 |
| Freitas & Mil-Homens (2015) | RCT (independent control group) | n = 10  Males (n = 10)  Females (n = 0) | n = 5 | n = 5 | 21.2 ± 0.8 | Healthy | Unclear | Not reported | Fascicle length | 4 | 4 |
| Gajdosik  (1991) | RCT (independent control group) | n = 24  Males (n = 24)  Females (n = 0) | n = 12 | n = 12 | IG = 25.3 ± 5  CG = 25.3 ± 5.6 | Healthy | Unclear | Limited | Maximum tolerable PRT;  Passive straight leg raise | 5 | 5 |
| Gajdosik et al. (2007) | RCT (independent control group) | n = 12  Males (n = 0)  Females (n = 12) | n = 6 | n = 4 | IG = 23 ± 4  CG = 21 ± 1 | Healthy | Sedentary | Not reported | Maximum tolerable PRT;  MTU stiffness;  Dorsiflexion passive ROM | 5 | 4 |
| Gajdosik et al. (2005) | RCT (independent control group) | n = 19  Males (n = 0)  Females (n = 19) | n = 10 | n = 9 | IG = 73.1 ± 6.8  CG = 75.3 ± 8.3 | Healthy | Sedentary and recreationally active | Limited | Maximum tolerable PRT;  MTU stiffness;  Dorsiflexion passive ROM | 6 | 6 |
| Halbertsma et al.  (1996) | RCT (independent control group) | n = 16  Males (n = 10)  Females (n = 6) | n = 10  Males (n = 6)  Females (n = 4) | n = 6  Males (n = 4)  Females (n = 2) | 24.2 ± 2.9  IG = 24.6 ± 3.3  CG = 23.7 ± 2.6 | Healthy | Unclear | Limited | Maximum tolerable PRT;  MTU stiffness;  Passive straight leg raise | 3 | 3 |
| Hatano et al.  (2022) | RCT (crossover study design) | n = 16  Males (n = 8)  Females (n = 8) | Crossover design |  | Males = 21.3 ± 0.7  Females = 20.9 ± 0.8 | Healthy | Sedentary and recreationally active | Not reported | Maximum tolerable PRT;  MTU stiffness;  Passive knee extension test | 4 | 4 |
| Herda et al. (2010) | RCT (crossover study design) | n = 11  Males (n = 11)  Females (n = 0) | Crossover design |  | 23 ± 2 | Healthy | Recreationally active | Not reported | MTU stiffness | 4 | 4 |
| Hunter et al. (2001) | RCT (independent control group) | n = 30  Males (n = 15)  Females (n = 15) | n = 15 | n = 15 | Males = 33.7 ± 6.8  Females = 40 ± 6.9 | Healthy | Recreationally active | Not reported | MTU stiffness | 4 | 4 |
| Ichihashi et al. (2016) | RCT (independent control group) | n = 30  Males (n = 30)  Females (n = 0) | n = 15 | n = 15 | 22.7 ± 2.2  IG = 22.5 ± 2.9  CG = 22.9 ± 1.2 | Healthy | Unclear | Not reported | Shear elastic modulus | 6 | 6 |
| Ikeda et al. (2021) | RCT (crossover study design) | n = 10  Males (n = 10)  Females (n = 0) | Crossover design |  | 25 ± 3 | Healthy | Unclear | Not reported | MTU stiffness;  Muscle stiffness;  Tendon stiffness;  Shear elastic modulus | 4 | 4 |
| Kaneda et al.  (2020) | CT (crossover study design) | n = 22  Males (n = 22)  Females (n = 0) | Crossover design |  | 22.5 ± 1 | Healthy | Recreationally active | Not reported | PRT at a given angle;  Maximum tolerable PRT;  Shear elastic modulus;  Fascicle length;  Dorsiflexion active ROM | 4 | 4 |
| Kay & Blazevich (2008) | RCT (crossover study design) | n = 7  Males (n = 4)  Females (n = 3) | Crossover design |  | 25.5 ± 5.3 | Healthy | Recreationally active | Not reported | PRT at a given angle | 4 | 4 |
| Konrad & Tilp (2020) | CT (independent control group) | n = 25  Males (n = 11)  Females (n = 14) | n = 11  Males (n = 4)  Females (n = 7) | n = 14  Males (n = 7)  Females (n = 7) | Males = 25.9 ± 6.9  Females = 24.1 ± 2.7 | Healthy | Trained | Not reported | PRT at a given angle;  MTU stiffness;  Muscle stiffness;  Tendon;  Dorsiflexion passive ROM | 1 | 1 |
| Konrad & Tilp (2014) | RCT (independent control group) | n = 49  Males (n = 35)  Females (n = 14) | n = 25 | n = 24 | Males = 23.3 ± 2.9  Females = 22.5 ± 2.5 | Healthy | Unclear (competitive athletes were excluded) | Not reported | PRT at a given angle;  MTU stiffness;  Muscle stiffness;  Tendon;  Fascicle length;  Dorsiflexion passive ROM | 2 | 2 |
| Konrad et al.  (2019) | RCT (crossover study design) | n = 14  Males (n = 7)  Females (n = 7) | Crossover design |  | Males = 27.5 ± 8.3  Females = 24.9 ± 3.1 | Healthy | Unclear | Not reported | PRT at a given angle;  MTU stiffness;  Muscle stiffness;  Tendon;  Dorsiflexion passive ROM | 4 | 4 |
| Konrad et al.  (2017) | CT (independent control group) | n = 122  Males (n = 79)  Females (n = 43) | n = 25  Males (n = 21)  Females (n = 4) | n = 24  Males (n = 11)  Females (n = 13) | Males = 23.3 ± 2.5  Females = 23.4 ± 3.7  IG = 23.3 ± 3.2  CG = 23.8 ± 3.5 | Healthy | Unclear (competitive athletes were excluded) | Not reported | PRT at a given angle;  MTU stiffness;  Muscle stiffness;  Tendon;  Fascicle length;  Dorsiflexion passive ROM | 3 | 3 |
| Krause et al.  (2019) | RCT (crossover study design) | n = 16  Males (n = 10)  Females (n = 6) | Crossover design |  | 32.1 ± 5 | Healthy | Unclear | Not reported | Maximum tolerable PRT;  MTU stiffness;  Knee flexion passive ROM | 4 | 4 |
| Kuruma et al. (2013) | RCT (independent control group) | n = 40  Males (n = 20)  Females (n = 20) | n = 10  Males (n = 5)  Females (n = 5) | n = 10  Males (n = 5)  Females (n = 5) | 21 (range = 19 to 24) | Healthy | Unclear | Not reported | Muscle stiffness;  Knee flexion passive ROM | 2 | 2 |
| Longo et al.  (2021) | RCT (independent control group) | n = 30  Males (n = 18)  Females (n = 12) | n = 15  Males (n = 9)  Females (n = 6) | n = 15  Males (n = 9)  Females (n = 6) | 22.7 ± 1.8  IG = 22.3 ± 0.8  CG = 23.4 ± 0.8 | Healthy | Recreationally active | Not reported | Maximum tolerable PRT;  MTU stiffness;  Muscle stiffness;  Fascicle length;  Dorsiflexion passive ROM | 5 | 5 |
| Madding et al. (1987) | RCT (independent control group) | n = 72  Males (n = 72)  Females (n = 0) | IG (1) n = 18  IG (2) n = 18  IG (3) n = 18 | n = 18 | 27.1 ± 4.4 (range = 22 to 40) | Healthy | Unclear | Limited | PRT at a given angle;  Hip abduction passive ROM | 4 | 4 |
| Maeda et al.  (2017) | RCT (crossover study design) | n = 20  Males (n = 20)  Females (n = 0) | Crossover design |  | 22.8 ± 1.4 | Healthy | Recreationally active | Not reported | Shear elastic modulus;  Dorsiflexion passive ROM | 4 | 4 |
| Mahieu et al. (2007) | RCT (independent control group) | n = 81  Males (n = 37)  Females (n = 44) | n = 31  Males (n = 21)  Females (n = 10) | n = 29  Males (n = 8)  Females (n = 21) | IG = 22 ± 1.1  CG = 22.3 ± 1.9 | Healthy | Recreationally active | Not reported | PRT at a given angle;  Tendon stiffness;  Dorsiflexion passive ROM | 6 | 5 |
| Marshall et al. (2011) | RCT (independent control group) | n = 22  Males (n = 14)  Females (n = 8) | n = 11 | n = 11 | 22.7 ± 3.8 | Healthy | Recreationally active | Not reported | Maximum tolerable PRT;  MTU stiffness;  Passive straight leg raise | 6 | 6 |
| Mizuno et al.  (2023) | RCT (crossover study design) | n = 16  Males (n = 7)  Females (n = 9) | Crossover design |  | 20.8 ± 0.8 | Healthy | Recreationally active | Not reported | PRT at a given angle;  Maximum tolerable PRT;  MTU stiffness;  Dorsiflexion passive ROM | 4 | 4 |
| Moltubakk et al. (2021) | RCT (contralateral extremity control group) | n = 26  Males (n = 9)  Females (n = 17) | Unilateral design |  | 22 ± 1.6 | Healthy | Recreationally active | Not reported | PRT at a given angle;  Maximum tolerable PRT;  Fascicle length;  Dorsiflexion passive ROM | 5 | 4 |
| Muir et al. (1999) | RCT (contralateral extremity control group) | n = 20  Males (n = 20)  Females (n = 0) | Unilateral design |  | 26.1 ± 4.2 | Healthy | Unclear | Not reported | PRT at a given angle | 5 | 5 |
| Murakami et al. (2024) | RCT (crossover study design) | n = 16  Males (n = 16)  Females (n = 0) | Crossover design |  | 21.4 ± 3.1 | Healthy | Sedentary | Not reported | Maximum tolerable PRT;  MTU stiffness;  Passive dorsiflexion ROM | 4 | 4 |
| Nakamura et al. (2017) | CT (independent control group) | n = 24  Males (n = 24)  Females (n = 0) | n = 12 | n = 12 | IG = 23.9 ± 3  CG = 23.6 ± 1 | Healthy | Recreationally active | Not reported | Maximum tolerable PRT;  MTU stiffness;  Dorsiflexion passive ROM | 3 | 3 |
| Nakamura et al. (2012) | RCT (independent control group) | n = 18  Males (n = 18)  Females (n = 0) | n = 9 | n = 9 | 21.4 ± 1.7  IG = 21.1 ± 2.3  CG = 21.8 ± 0.8 | Healthy | Unclear | Not reported | PRT at a given angle;  Fascicle length;  Dorsiflexion passive ROM | 4 | 4 |
| Nakamura et al. (2021a) | RCT (independent control group) | n = 40  Males (n = 40)  Females (n = 0) | IG (1) n = 14  IG (2) n = 13 | n = 13 | IG (1) = 21.4 ± 1  IG (2) = 21.4 ± 1.1  CG = 21.9 ± 1.3 | Healthy | Unclear | Not reported | Maximum tolerable PRT;  Muscle stiffness;  Fascicle length;  Dorsiflexion passive ROM | 4 | 4 |
| Nakamura et al. (2021b) | CT (crossover study design) | n = 15  Males (n = 15)  Females (n = 0) | Crossover design |  | 21.5 ± 1.5 | Healthy | “None of the participants were competitive athletes or engaged in regular resistance training or stretching programs for the lower limbs” | Not reported | Maximum tolerable PRT;  Muscle stiffness;  Dorsiflexion passive ROM | 3 | 3 |
| Nakao et al. (2021) | CT (independent control group) | n = 30  Males (n = 30)  Females (n = 0) | n = 15 | n = 15 | 22.7 ± 2.2  IG = 22.5 ± 2.9  CG = 22.9 ± 1.2 | Healthy | Recreationally active | Not reported | MTU stiffness | 4 | 4 |
| Oba et al.  (2021) | RCT (crossover study design) | n = 14  Males (n = 14)  Females (n = 0) | Crossover design |  | 22.9 ± 1 (range = 22 to 25) | Healthy | Unclear | Not reported | MTU stiffness;  Dorsiflexion passive ROM | 5 | 5 |
| O’Connor et al. (2009) | CT (contralateral extremity control group) | n = 10  Males (n = 10)  Females (n = 0) | Unilateral design |  | 20.6 ± 0.3 | Healthy | Unclear | Not reported | Fascicle length | 3 | 3 |
| Opplert et al. (2019) | RCT (crossover study design) | n = 13  Males (n = 13)  Females (n = 0) | Crossover design |  | 24.9 ± 2.5 | Healthy | Recreationally active | Not reported | PRT at a given angle;  Fascicle length | 4 | 4 |
| Palmer et al.  (2022) | RCT (crossover study design) | n = 15  Males (n = 15)  Females (n = 0) | Crossover design |  | 70 ± 7 | Healthy | Recreationally active | Not reported | MTU stiffness;  Passive straight leg raise | 4 | 4 |
| Palmer et al.  (2019) | RCT (crossover study design) | n = 13  Males (n = 0)  Females (n = 13) | Crossover design |  | 21 ± 2 (range = 18 to 25) | Healthy | Trained | Limited | MTU stiffness;  Passive straight leg raise | 4 | 4 |
| Palmer et al.  (2018) | RCT (crossover study design) | n = 11  Males (n = 11)  Females (n = 0) | Crossover design |  | 69 ± 6 | Healthy | Recreationally active | Not reported | MTU stiffness | 5 | 5 |
| Peixinho et al. (2016) | RCT (independent control group) | n = 16  Males (n = 16)  Females (n = 0) | n = 8 | n = 8 | 18.7 ± 0.4 | Healthy | Recreationally active | Not reported | Maximum tolerable PRT;  Dorsiflexion passive ROM | 3 | 3 |
| Peixinho et al. (2021) | RCT (independent control group) | n = 20  Males (n = 20)  Females (n = 0) | n = 12 | n = 8 | 18.9 ± 0.5 | Healthy | Recreationally active | Not reported | Maximum tolerable PRT;  Tendon stiffness;  Fascicle length;  Dorsiflexion passive ROM | 6 | 5 |
| Rihvk et al. (2010) | CT (independent control group) | n = 30  Males (n = 30)  Females (n = 0) | n = 10 | n = 10 | 22.6 ± 3.3  IG = 22.8 ± 3.8  CG = 23.1 ± 3.2 | Healthy | Athletes | Not reported | PRT at a given angle | 2 | 2 |
| Rodrigues et al. (2017) | CT (crossover study design) | n = 12  Males (n = 12)  Females (n = 0) | Crossover design |  | 22.3 ± 1.9 | Healthy | Participants were “not involved in any activity involving flexibility and strength training for lower limbs in the last 12 months” | Not reported | PRT at a given angle;  Maximum tolerable PRT;  MTU stiffness;  Dorsiflexion passive ROM | 2 | 2 |
| Ryan (2009) | RCT (independent control group) | n = 26  Males (n = 26)  Females (n = 0) | n = 15 | n = 11 | IG = 22 ± 2  CG = 21 ± 2 | Healthy | Trained | Not reported | MTU stiffness;  Dorsiflexion passive ROM | 4 | 4 |
| Ryan et al. (2008) | RCT (crossover study design) | n = 12  Males (n = 7)  Females (n = 5) | Crossover design |  | 24 ± 3 | Healthy | Recreationally active | Not reported | MTU stiffness | 4 | 4 |
| Sá et al. (2016) | RCT (crossover study design) | n = 9  Males (n = 9)  Females (n = 0) | Crossover design |  | 24.8 ± 3 | Healthy | Recreationally active | Limited | Fascicle length | 6 | 5 |
| Şekir et al. (2019) | RCT (independent control group) | n = 34  Males (n = 34)  Females (n = 0) | n = 12 | n = 11 | IG = 23.1 ± 3.1  CG = 22.2 ± 2.9 | Healthy | Recreationally active | Not reported | Fascicle length | 8 | 7 |
| Sonda et al.  (2022) | RCT (independent control group) | n = 30  Males (n = 10)  Females (n = 20) | n = 15  Males (n = 4)  Females (n = 11) | n = 15  Males (n = 6)  Females (n = 9) | 26.6 ± 3.8  IG = 26.7 ± 3.2  CG = 26.5 ± 4.5 | Healthy | Sedentary | Not reported | PRT at a given angle;  MTU stiffness;  Muscle stiffness;  Tendon stiffness;  Fascicle length;  Dorsiflexion passive ROM | 6 | 5 |
| Stafilidis et al. (2015) | RCT (crossover study design) | n = 11  Males (n = 8)  Females (n = 3) | Crossover design |  | 25.5 ± 3.1 | Healthy | Trained | Not reported | PRT at a given angle;  MTU stiffness;  Knee flexion passive ROM | 4 | 4 |
| Umehara et al. (2018) | CT (contralateral extremity control group) | n = 20  Males (n = 20)  Females (n = 0) | Unilateral design |  | 25.4 ± 3.1 | Healthy | Unclear | Not reported | Shear elastic modulus | 4 | 3 |
| Viera et al. (2021) | RCT (crossover study design) | n = 13  Males (n = 13)  Females (n = 0) | Crossover design |  | 24.9 ± 2.5 | Healthy | Recreationally active | Not reported | PRT at a given angle;  Muscle stiffness;  Fascicle length | 4 | 4 |
| Warneke et al. (2024) | RCT (crossover study design) | n = 40  Males (n = 25)  Females (n = 15) | Crossover design |  | 27.7 ± 5.5 | Healthy | Trained | Not reported | Shear elastic modulus; Passive dorsiflexion ROM | 6 | 6 |
| Wiemann & Kahn (1997) | RCT (independent control group) | n = 69  Males (n = 69)  Females (n = 0) | n = 14 | n = 15 | Range = 20 to 34 | Healthy | Unclear | Not reported | Maximum tolerable PRT;  Passive*  straight leg raise | 3 | 3 |
| Yahata et al. (2021) | CT (contralateral extremity control group) | n = 16  Males (n = 16)  Females (n = 0) | Unilateral design |  | 21.4 ± 1.5 | Healthy | Unclear | Not reported | Fascicle length | 4 | 4 |
