# Supplementary material for: Mechanisms Underlying Range of Motion Improvements Following Acute and Chronic Static Stretching: A Systematic Review, Meta-analysis and Multivariate Meta-regression
Source: Sports Med. 2025 Apr 3;55(6):1449–66. doi: 10.1007/s40279-025-02204-7 (PMC12152101; doi:10.1007/s40279-025-02204-7)
Supplement: Supplementary file 4 — Supplementary file4 (DOCX 29 KB) [file 40279_2025_2204_MOESM4_ESM.docx]

**Title:** Mechanisms Underlying Range of Motion Improvements Following Acute and Chronic Static Stretching: A Systematic Review, Meta-Analysis, and Multivariate Meta-Regression

**Journal Name:** Sports Medicine

**Authors:** Lewis Ingram^1^, Grant Tomkinson^1^, Noah D’Unienville^1^, Bethany Gower^1^, Sam Gleadhill^1^, Terry Boyle^2^, and Hunter Bennett^1^

**Affiliations:**

^1^Alliance for Research in Exercise, Nutrition and Activity (ARENA), Allied Health and Human Performance, University of South Australia, Adelaide, SA, Australia

^2^Australian Centre for Precision Health, Allied Health and Human Performance, University of South Australia, Adelaide, SA, Australia

**Corresponding author**

Lewis Ingram

Email: [Lewis.Ingram@unisa.edu.au](mailto:Lewis.Ingram@unisa.edu.au)

**Table S3** Characteristics of acute static stretching interventions

| **Study** | **Stretched muscles** | **Side of stretch performed** | **Number of exercises** | **Number of sets** | **Stretching duration (per set) (s)** | **Total stretching duration (min)** | **Stretching intensity** |
| --- | --- | --- | --- | --- | --- | --- | --- |
| Barbosa et al. (2018) | Hamstrings (unilateral) | Single-side stretch performed on each side | 1 | 3 | 30 | 1.5 | Moderate |
| Cannavan et al. (2012) | Ankle plantarflexors (unilateral) | Single-side stretch performed, unclear whether or not both sides were stretched | 1 | 4 | 45 | 3 | High |
| de Oliveira et al. (2018) | Hamstrings (unilateral) | Unclear which side was stretched | 1 | 1 | 180 | 3 | Low |
| Farrow et al. (2024) | Hamstrings (right side) | Right side only | 1 | 4 | 15 | 1 | Moderate |
| Halbertsma et al.  (1996) | Hamstrings (unilateral) | Single-side stretch performed, unclear whether or not both sides were stretched | 1 | 10 | 30 | 5 | Not reported |
| Hatano et al.  (2022) | Hamstrings (right side) | Right side only | 1  1  1 | 1  1  1 | 300  300  300 | 5  5  5 | High (100% intensity  High (110% intensity)  High (120% intensity) |
| Herda et al. (2010) | Ankle plantarflexors (right side) | Right side only | 1 | 9 | 135 | 20.25 | Moderate |
| Hunter et al. (2001) | Ankle plantarflexors  (bilateral) | Both sides stretched simultaneously | 1 | 10 | 30 | 5 | Moderate |
| Ikeda et al. (2021) | Ankle plantarflexors [gastrocnemius] (unilateral) | Unclear which side was stretched | 1 | 5 | 60 | 5 | High |
| Kaneda et al.  (2020) | Ankle plantarflexors [gastrocnemius] (unilateral) | Non-dominant side only | 1 | 5 | 60 | 5 | High |
| Kay & Blazevich (2008) | Ankle plantarflexors [soleus] (unilateral) | Unclear which side was stretched | 1  1  1  1 | 1  1  4  4 | 5  15  5  15 | 0.083  0.25  0.33  1 | High  High  High  High |
| Konrad & Tilp (2020) | Ankle plantarflexors [gastrocnemius] | Unclear which side (or both sides) was stretched, or whether the stretch itself was unilateral or bilateral | 1 | 2 | 30 | 1 | High |
| Konrad et al.  (2019) | Ankle plantarflexors (unilateral) | Unclear which side was stretched | 1 | 5 | 60 | 5 | High |
| Konrad et al.  (2017) | Ankle plantarflexors | Unclear which side (or both sides) was stretched, or whether the stretch itself was unilateral or bilateral | 1 | 4 | 30 | 2 | High |
| Krause et al.  (2019) | Quadriceps | Unclear which side (or both sides) was stretched, or whether the stretch itself was unilateral or bilateral | 1 | 2 | 60 | 2 | Moderate |
| Kuruma et al. (2013) | Quadriceps | Unclear which side (or both sides) was stretched, or whether the stretch itself was unilateral or bilateral | 1 | 1 | 480 | 8 | Not reported |
| Madding et al. (1987) | Hip adductors (unilateral) | Left side only | 1  1  1 | 1  1  1 | 15  45  120 | 0.25  0.75  2 | Moderate  Moderate  Moderate |
| Maeda et al.  (2017) | Ankle plantarflexors [gastrocnemius] (unilateral) | Non-dominant (left) side only | 1 | 1 | 120 | 2 | High |
| Muir et al. (1999) | Ankle plantarflexors [gastrocnemius] (unilateral) | Side stretched was randomised | 1 | 4 | 30 | 2 | Low |
| Murakami et al. (2024) | Ankle plantarflexors [gastrocnemius] (unilateral) | Dominant side only | 1 | 3 | 60 | 3 | Low |
| Oba et al.  (2021) | Ankle plantarflexors (unilateral) | Right side only | 1  1  1 | 5  5  5 | 60  60  60 | 5  5  5 | Moderate  Low  Low |
| Opplert et al. (2019) | Ankle plantarflexors (unilateral) | Right side only | 1 | 2 | 15 | 0.5 | High |
| Palmer et al.  (2022) | Hamstrings (unilateral) | Right side only | 1 | 4 | 15 | 1 | Moderate |
| Palmer et al.  (2019) | Hamstrings (unilateral) | Right side only | 1  1  1 | 1  2  4 | 30  30  30 | 0.5  1  2 | Moderate  Moderate  Moderate |
| Palmer et al.  (2018) | Hamstrings (unilateral) | Right side only | 1 | 4 | 15 | 1 | Moderate |
| Rihvk et al. (2010) | Hamstrings (unilateral) | Dominant side only | 1 | 1 | 180 | 3 | High |
| Rodrigues et al. (2017) | Hamstrings (unilateral) | Right side only | 1 | 4 | 30 | 2 | High |
| Ryan et al. (2008) | Ankle plantarflexors [gastrocnemius] (unilateral) | Right side only | 1  1  1 | 4  8  16 | 30  30  30 | 2  4  8 | Moderate  Moderate  Moderate |
| Sá et al. (2016) | Quadriceps and hamstrings (unilateral) | Single-side stretch performed, unclear which side or whether or not both sides were stretched | 2 | 3 | 30 | 3 | Moderate |
| Şekir et al. (2019) | Ankle evertors and dorsiflexors (unilateral) | Dominant side only | 3 | 4 | 30 | 6 | Moderate |
| Sonda et al.  (2022) | Ankle plantarflexors (unilateral) | Side stretched was randomised | 1 | 1 | 600 | 10 | Moderate |
| Stafilidis et al. (2015) | Quadriceps, hamstrings, and ankle plantarflexors (unilateral) | Single-side stretches performed on each side | 3  5 | 1  1 | 15  60 | 0.75  3 | Moderate |
| Umehara et al. (2018) | Pectoralis minor (unilateral) | Non-dominant side only | 1 | 10 | 30 | 5 | Moderate |
| Viera et al. (2021) | Ankle plantarflexors (unilateral) | Right side only | 1 | 2 | 15 | 0.5 | High |
| Warneke et al. (2024) | Ankle plantarflexors [gastrocnemius] (unilateral) | Single side stretch, unclear which side was stretched | 1 | 1 | 300 | 5 | High |
| Wiemann & Kahn (1997) | Hamstrings | Unclear which side (or both sides) was stretched, or whether the stret3ches themselves were unilateral or bilateral | 3 | 3 | 15 | 2.3 | Not reported |
