# Supplementary material for: Mechanisms Underlying Range of Motion Improvements Following Acute and Chronic Static Stretching: A Systematic Review, Meta-analysis and Multivariate Meta-regression
Source: Sports Med. 2025 Apr 3;55(6):1449–66. doi: 10.1007/s40279-025-02204-7 (PMC12152101; doi:10.1007/s40279-025-02204-7)
Supplement: Supplementary file 5 — Supplementary file5 (DOCX 30 KB) [file 40279_2025_2204_MOESM5_ESM.docx]

**Title:** Mechanisms Underlying Range of Motion Improvements Following Acute and Chronic Static Stretching: A Systematic Review, Meta-Analysis, and Multivariate Meta-Regression

**Journal Name:** Sports Medicine

**Authors:** Lewis Ingram^1^, Grant Tomkinson^1^, Noah D’Unienville^1^, Bethany Gower^1^, Sam Gleadhill^1^, Terry Boyle^2^, and Hunter Bennett^1^

**Affiliations:**

^1^Alliance for Research in Exercise, Nutrition and Activity (ARENA), Allied Health and Human Performance, University of South Australia, Adelaide, SA, Australia

^2^Australian Centre for Precision Health, Allied Health and Human Performance, University of South Australia, Adelaide, SA, Australia

**Corresponding author**

Lewis Ingram

Email: [Lewis.Ingram@unisa.edu.au](mailto:Lewis.Ingram@unisa.edu.au)

**Table S4** Characteristics of chronic static stretching interventions

| **Study** | **Stretched muscles** | **Side of stretch performed** | **Study duration (weeks)** | **Frequency per week** | **Number of exercises** | **Number of sets** | **Stretching duration (per set) (s)** | **Stretch duration (per session) (min)** | **Weekly stretch duration (min)** | **Total stretching duration (min)** | **Stretching intensity** |
| --- | --- | --- | --- | --- | --- | --- | --- | --- | --- | --- | --- |
| Akagi & Takahashi (2014) | Ankle plantarflexors (unilateral) | Side stretched was randomised | 5 | 6 | 1 | 3 | 120 | 6 | 36 | 180 | Low |
| Andrade et al. (2020) | Ankle plantarflexors (bilateral) | Both sides stretched in single stretch | 12 | 5 | 2 | 5 | 45 | 7.5 | 37.5 | 900 | High |
| Aquino et al. (2010) | Hamstrings (unilateral) | Single-side stretch performed on each side | 8 | 3 | 1 | 4 | 30 | 2 | 6 | 48 | Moderate |
| Barbosa et al. (2018) | Hamstrings (unilateral) | Single-side stretch performed on each side | 3.3 | 3 | 1 | 3 | 30 | 1.5 | 4.5 | 15 | Moderate |
| Ben & Harvey (2010) | Hamstrings (unilateral) | Side stretched was randomised | 6 | 5 | 1 | 1 | 1800 | 30 | 150 | 900 | High |
| Blazevich et al. (2014) | Ankle plantarflexors (unilateral) | Unclear which side was stretched | 3 | 14 | 1 | 4 | 30 | 2 | 28 | 84 | High |
| Cini et al. (2024) | Ankle plantarflexors [gastrocnemius] (unilateral) | Single-side stretch performed on each side | 6  6 | 3  3 | 1  1 | 1  1 | 120  300 | 2  5 | 6  15 | 36  90 | High  High |
| e Lima et al.  (2015) | Knee extensors, hamstrings (unilateral) | Single-side stretch performed, unclear whether or not both sides were stretched | 8 | 3 | 2 | 3 | 30 | 3 | 9 | 72 | Moderate |
| Folpp et al. (2006) | Hamstrings (unilateral) | Side stretched was randomised | 4 | 5 | 1 | 1 | 1200 | 20 | 100 | 400 | High |
| Freitas & Mil-Homens (2015) | Hamstrings (unilateral) | Single-side stretch performed, unclear whether or not both sides were stretched | 8 | 5 | 1 | 1 | 450 | 7.5 | 37.5 | 300 | Moderate |
| Gajdosik  (1991) | Hamstrings (unilateral) | Single-side stretch performed on each side | 3 | 7 | 1 | 10 | 15 | 2.5 | 17.5 | 52.5 | High |
| Gajdosik et al. (2007) | Ankle plantarflexors (unilateral) | Single-side stretch performed on each side | 6 | 5 | 1 | 10 | 15 | 2.5 | 12.5 | 75 | Not reported |
| Gajdosik et al. (2005) | Ankle plantarflexors (unilateral) | Single-side stretch performed on each side | 8 | 3 | 1 | 10 | 15 | 2.5 | 7.5 | 60 | Not reported |
| Ichihashi et al. (2016) | Hamstrings (unilateral) | Dominant side only | 4 | 3 | 1 | 1 | 300 | 5 | 15 | 60 | Low |
| Konrad & Tilp (2014) | Ankle plantarflexors [gastrocnemius] (unilateral) | Single-side stretch performed on each side | 6 | 5 | 1 | 4 | 30 | 2 | 10 | 60 | Moderate |
| Longo et al.  (2021) | Ankle plantarflexors [gastrocnemius] (unilateral) | Dominant (right) side only | 12 | 5 | 2 | 5 | 45 | 7.5 | 37.5 | 450 | High |
| Mahieu et al. (2007) | Ankle plantarflexors (unilateral) | Single-side stretch performed on each side | 6 | 7 | 1 | 5 | 20 | 1.7 | 11.7 | 70 | Moderate |
| Marshall et al. (2011) | Hamstrings and gluteals (unilateral and bilateral) | Three (3) single side stretches performed on each side, one (1) bilateral stretch | 4 | 5 | 4 | 3 | 30 | 6 | 30 | 120 | Not reported |
| Mizuno (2023) | Ankle plantarflexors (bilateral) | Both sides stretched simultaneously | 8 | 3 | 1 | 4 | 30 | 2 | 6 | 48 | Moderate |
| Moltubakk et al. (2021) | Ankle plantarflexors [gastrocnemius and soleus] (unilateral) | Right side only | 24 | 7 | 1 | 4 | 60 | 4 | 28 | 672 | Moderate |
| Nakamura et al. (2017) | Ankle plantarflexors (unilateral) | Single-side stretch performed, unclear which sides was stretched | 4 | 3 | 1 | 4 | 30 | 2 | 6 | 24 | Not reported |
| Nakamura et al. (2012) | Ankle plantarflexors [gastrocnemius]  (bilateral) | Both sides stretched simultaneously | 4 | 7 | 1 | 2 | 60 | 2 | 14 | 56 | High |
| Nakamura et al. (2021a) | Ankle plantarflexors [gastrocnemius]  (bilateral) | Both sides stretched simultaneously | 4  4 | 3  3 | 1  1 | 3  3 | 60  60 | 3  3 | 12  12 | 36  36 | Moderate  Low |
| Nakamura et al. (2021b) | Ankle plantarflexors (unilateral) | Dominant side only | 5 | 2 | 1 | 6 | 300 | 30 | 60 | 300 | High |
| Nakao et al. (2021) | Hamstrings (unilateral) | Dominant side only | 4 | 3 | 1 | 1 | 300 | 5 | 15 | 60 | Moderate |
| O’Connor et al. (2009) | Ankle plantarflexors [gastrocnemius]  (unilateral) | Right side only | 1  1 | 4  8 | 1 | 1  2 | 30  30 | 0.5  1 | 2  4 | 2  4 | Moderate  Moderate |
| Peixinho et al. (2016) | Ankle plantarflexors (unilateral) | Single-side stretch performed on each side | 10 | 4.5 | 2 | 2 | 30 | 2 | 9 | 90 | High |
| Peixinho et al. (2021) | Ankle plantarflexors (unilateral) | Single-side stretch performed on each side | 10 | 4.5 | 2 | 2 | 30 | 2 | 9 | 90 | Moderate |
| Ryan (2009) | Ankle plantarflexors (unilateral) | Right side only | 4 | 3 | 1 | 4 | 135 | 9 | 27 | 108 | Moderate |
| Şekir et al. (2019) | Ankle evertors and dorsiflexors (unilateral) | Dominant side only | 6 | 5 | 3 | 4 | 30 | 6 | 30 | 180 | Moderate |
| Yahata et al. (2021) | Ankle plantarflexors (unilateral) | Dominant side only | 5 | 2 | 1 | 6 | 300 | 30 | 60 | 300 | High |
