# Supplementary material for: Mechanisms Underlying Range of Motion Improvements Following Acute and Chronic Static Stretching: A Systematic Review, Meta-analysis and Multivariate Meta-regression
Source: Sports Med. 2025 Apr 3;55(6):1449–66. doi: 10.1007/s40279-025-02204-7 (PMC12152101; doi:10.1007/s40279-025-02204-7)
Supplement: Supplementary file 6 — Supplementary file6 (DOCX 38 KB) [file 40279_2025_2204_MOESM6_ESM.docx]

**Title:** Mechanisms Underlying Range of Motion Improvements Following Acute and Chronic Static Stretching: A Systematic Review, Meta-Analysis, and Multivariate Meta-Regression

**Journal Name:** Sports Medicine

**Authors:** Lewis Ingram^1^, Grant Tomkinson^1^, Noah D’Unienville^1^, Bethany Gower^1^, Sam Gleadhill^1^, Terry Boyle^2^, and Hunter Bennett^1^

**Affiliations:**

^1^Alliance for Research in Exercise, Nutrition and Activity (ARENA), Allied Health and Human Performance, University of South Australia, Adelaide, SA, Australia

^2^Australian Centre for Precision Health, Allied Health and Human Performance, University of South Australia, Adelaide, SA, Australia

**Corresponding author**

Lewis Ingram

Email: [Lewis.Ingram@unisa.edu.au](mailto:Lewis.Ingram@unisa.edu.au)

**Table S5** Risk of Bias assessment

| **Authors** | **Eligibility criteria** | **Random allocation** | **Concealed allocation** | **Baseline comparability** | **Blind subjects** | **Blind therapists** | **Blind assessors** | **Adequate follow-up** | **Intention-to-treat analysis** | **Between-group comparisons** | **Point estimates and variability** | **Score** | **Adjusted PEDro score** |
| --- | --- | --- | --- | --- | --- | --- | --- | --- | --- | --- | --- | --- | --- |
| Akagi & Takahashi (2014) | No | Yes | No | Yes | No | No | No | No | Yes | Yes | Yes | 5 | 5 |
| Andrade et al. (2020) | Yes | Yes | Yes | Yes | No | No | No | Yes | No | Yes | Yes | 6 | 6 |
| Aquino et al. (2010) | Yes | Yes | No | No | No | No | Yes | No | No | Yes | Yes | 4 | 3 |
| Barbosa et al. (2018) | Yes | Yes | No | Yes | No | No | Yes | No | No | Yes | Yes | 5 | 4 |
| Ben & Harvey (2010) | Yes | Yes | Yes | Yes | No | No | Yes | Yes | Yes | Yes | Yes | 8 | 7 |
| Blazevich et al. (2014) | No | Yes | Yes | No | No | No | No | Yes | No | Yes | Yes | 5 | 5 |
| Cannavan et al. (2012) | No | No | No | Yes | No | No | No | No | Yes | No | No | 2 | 2 |
| Cini et al. (2024) | Yes | Yes | No | Yes | No | No | Yes | Yes | Yes | Yes | Yes | 7 | 6 |
| de Oliveira et al. (2018) | Yes | Yes | No | No | No | No | No | No | No | Yes | Yes | 3 | 3 |
| e Lima et al. (2015) | No | Yes | No | Yes | No | No | Yes | No | No | Yes | Yes | 5 | 4 |
| Farrow et al. (2024) | No | Yes | No | Yes | No | No | No | No | No | Yes | Yes | 4 | 4 |
| Folpp et al. (2006) | Yes | Yes | Yes | Yes | No | No | Yes | Yes | Yes | Yes | Yes | 8 | 7 |
| Freitas & Mil-Homens (2015) | No | Yes | No | Yes | No | No | No | Yes | No | No | Yes | 4 | 4 |
| Gajdosik (1991) | No | Yes | No | Yes | No | No | No | Yes | No | Yes | Yes | 5 | 5 |
| Gajdosik et al. (2007) | No | Yes | No | Yes | No | No | Yes | No | No | Yes | Yes | 5 | 4 |
| Gajdosik et al. (2005) | Yes | Yes | Yes | Yes | No | No | No | Yes | No | Yes | Yes | 6 | 6 |
| Halbertsma et al. (1996) | Yes | Yes | No | No | No | No | No | No | No | Yes | Yes | 3 | 3 |
| Hatano et al. (2022) | Yes | Yes | No | Yes | No | No | No | No | No | Yes | Yes | 4 | 4 |
| Herda et al. (2010) | No | Yes | No | Yes | No | No | No | No | No | Yes | Yes | 4 | 4 |
| Hunter et al. (2001) | No | Yes | No | No | No | No | No | Yes | No | Yes | Yes | 4 | 4 |
| Ichihashi et el. (2016) | Yes | Yes | No | Yes | No | No | No | Yes | Yes | Yes | Yes | 6 | 6 |
| Ikeda et al. (2021) | No | Yes | No | Yes | No | No | No | No | No | Yes | Yes | 4 | 4 |
| Kaneda et al. (2020) | Yes | No | No | Yes | No | No | No | Yes | No | Yes | Yes | 4 | 4 |
| Kay & Blazevich (2008) | No | Yes | No | Yes | No | No | No | No | No | Yes | Yes | 4 | 4 |
| Konrad & Tilp (2020) | No | No | No | No | No | No | No | No | No | No | Yes | 1 | 1 |
| Konrad & Tilp (2014) | Yes | Yes | No | No | No | No | No | No | No | No | Yes | 2 | 2 |
| Konrad et al. (2019) | No | Yes | No | Yes | No | No | No | No | No | Yes | Yes | 4 | 4 |
| Konrad et al. (2017) | Yes | No | No | No | No | No | No | Yes | No | Yes | Yes | 3 | 3 |
| Krause et al. (2019) | Yes | Yes | No | Yes | No | No | No | No | No | Yes | Yes | 4 | 4 |
| Kuruma et al. (2013) | No | Yes | No | No | No | No | No | No | No | Yes | No | 2 | 2 |
| Longo et al. (2021) | Yes | Yes | No | Yes | No | No | No | Yes | No | Yes | Yes | 5 | 5 |
| Madding et al. (1987) | Yes | Yes | No | No | No | No | No | Yes | No | Yes | Yes | 4 | 4 |
| Maeda et al. (2017) | Yes | Yes | No | Yes | No | No | No | No | No | Yes | Yes | 4 | 4 |
| Mahieu et al. (2007) | Yes | Yes | Yes | Yes | No | No | Yes | No | No | Yes | Yes | 6 | 5 |
| Marshall et al. (2011) | Yes | Yes | No | Yes | No | No | No | Yes | Yes | Yes | Yes | 6 | 6 |
| Mizuno et al. (2023) | No | Yes | No | Yes | No | No | No | No | No | Yes | Yes | 4 | 4 |
| Moltubakk et al. (2021) | Yes | Yes | No | Yes | No | No | Yes | No | No | Yes | Yes | 5 | 4 |
| Muir et al. (1999) | Yes | Yes | No | Yes | No | No | No | Yes | No | Yes | Yes | 5 | 5 |
| Murakami et al. (2024) | Yes | Yes | No | Yes | No | No | No | No | No | Yes | Yes | 4 | 4 |
| Nakamura et al. (2017) | Yes | No | No | Yes | No | No | No | No | No | Yes | Yes | 3 | 3 |
| Nakamura et al. (2012) | Yes | Yes | No | Yes | No | No | No | No | No | Yes | Yes | 4 | 4 |
| Nakamura et al. (2021a) | Yes | Yes | No | Yes | No | No | No | No | No | Yes | Yes | 4 | 4 |
| Nakamura et al. (2021b) | Yes | No | No | Yes | No | No | No | No | No | Yes | Yes | 3 | 3 |
| Nakao et al. (2021) | No | No | No | No | No | No | No | Yes | Yes | Yes | Yes | 4 | 4 |
| Oba et al. (2021) | No | Yes | Yes | Yes | No | No | No | No | No | Yes | Yes | 5 | 5 |
| O’Connor et al. (2009) | Yes | No | No | Yes | No | No | No | Yes | No | Yes | No | 3 | 3 |
| Opplert et al. (2019) | No | Yes | No | Yes | No | No | No | No | No | Yes | Yes | 4 | 4 |
| Palmer et al. (2022) | No | Yes | No | Yes | No | No | No | No | No | Yes | Yes | 4 | 4 |
| Palmer et al. (2019) | No | Yes | No | Yes | No | No | No | No | No | Yes | Yes | 4 | 4 |
| Palmer et al. (2018) | No | Yes | No | Yes | No | No | No | Yes | No | Yes | Yes | 5 | 5 |
| Peixinho et al. (2016) | No | Yes | No | No | No | No | No | No | No | Yes | Yes | 3 | 3 |
| Peixinho et al. (2021) | No | Yes | No | Yes | No | No | Yes | Yes | No | Yes | Yes | 6 | 5 |
| Rihvk et al. (2010) | Yes | No | No | Yes | No | No | No | No | No | Yes | No | 2 | 2 |
| Rodrigues et al. (2017) | No | No | No | Yes | No | No | No | No | No | No | Yes | 2 | 2 |
| Ryan (2009) | No | Yes | No | Yes | No | No | No | No | No | Yes | Yes | 4 | 4 |
| Ryan et al. (2008) | No | Yes | No | Yes | No | No | No | No | No | Yes | Yes | 4 | 4 |
| Sá et al. (2016) | Yes | Yes | No | Yes | No | No | Yes | Yes | No | Yes | Yes | 6 | 5 |
| Şekir et al. (2019) | Yes | Yes | Yes | Yes | No | No | Yes | Yes | Yes | Yes | Yes | 8 | 7 |
| Sonda et al. (2022) | Yes | Yes | Yes | Yes | No | No | Yes | No | No | Yes | Yes | 6 | 5 |
| Stafilidis et al. (2015) | No | Yes | No | Yes | No | No | No | No | No | Yes | Yes | 4 | 4 |
| Umehara et al. (2018) | Yes | No | No | Yes | No | No | Yes | No | No | Yes | Yes | 4 | 3 |
| Vieira et al. (2021) | No | Yes | No | Yes | No | No | No | No | No | Yes | Yes | 4 | 4 |
| Warneke et al. (2024) | Yes | Yes | No | Yes | No | No | No | Yes | Yes | Yes | Yes | 6 | 6 |
| Wiemann & Kahn (1997) | No | Yes | No | No | No | No | No | No | No | Yes | Yes | 3 | 3 |
| Yahata et al. (2021) | Yes | No | No | Yes | No | No | No | No | Yes | Yes | Yes | 4 | 4 |
