# Supplementary material for: Mechanisms Underlying Range of Motion Improvements Following Acute and Chronic Static Stretching: A Systematic Review, Meta-analysis and Multivariate Meta-regression
Source: Sports Med. 2025 Apr 3;55(6):1449–66. doi: 10.1007/s40279-025-02204-7 (PMC12152101; doi:10.1007/s40279-025-02204-7)

**Title:** Mechanisms Underlying Range of Motion Improvements Following Acute and Chronic Static Stretching: A Systematic Review, Meta-Analysis, and Multivariate Meta-Regression

**Journal Name:** Sports Medicine

**Authors:** Lewis Ingram^1^, Grant Tomkinson^1^, Noah D’Unienville^1^, Bethany Gower^1^, Sam Gleadhill^1^, Terry Boyle^2^, and Hunter Bennett^1^

**Affiliations:**

^1^Alliance for Research in Exercise, Nutrition and Activity (ARENA), Allied Health and Human Performance, University of South Australia, Adelaide, SA, Australia

^2^Australian Centre for Precision Health, Allied Health and Human Performance, University of South Australia, Adelaide, SA, Australia

**Corresponding author**

Lewis Ingram

Email: [Lewis.Ingram@unisa.edu.au](mailto:Lewis.Ingram@unisa.edu.au)

Forest plot for chronic SS studies on maximum tolerable PRT


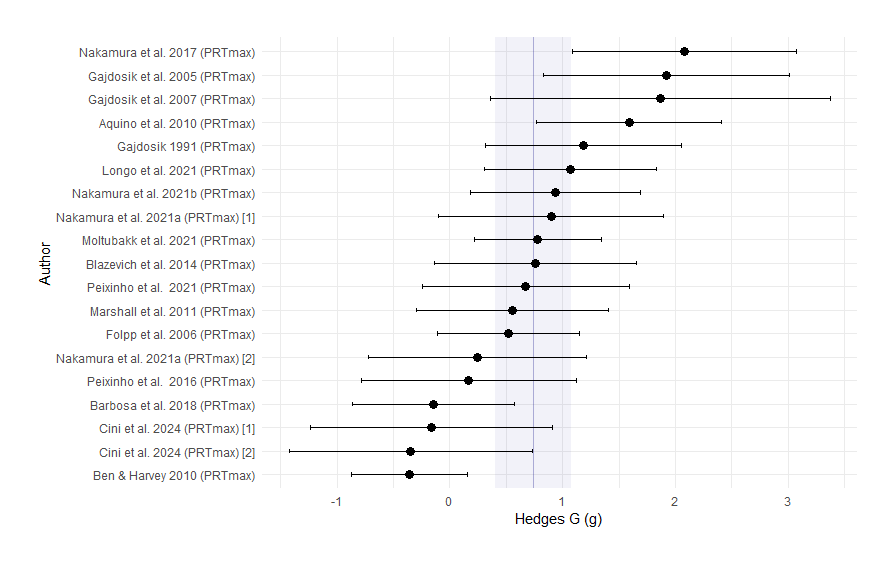


Forest plot for chronic SS studies on overall stiffness


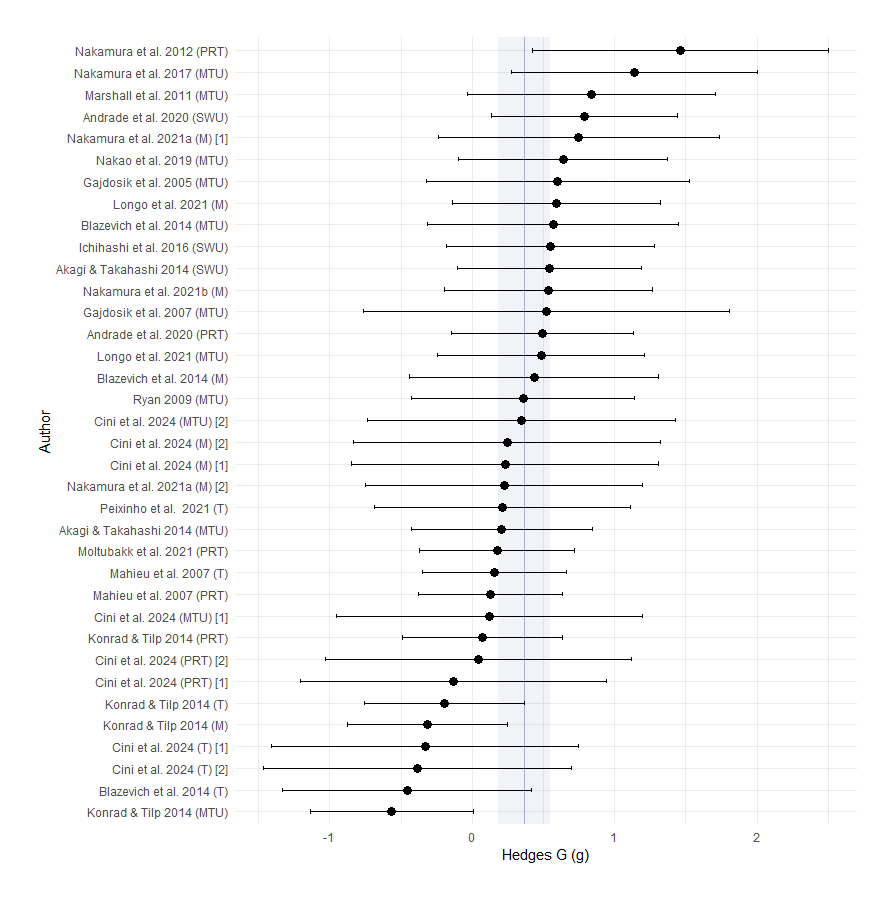


Forest plot for chronic SS studies on fascicle length


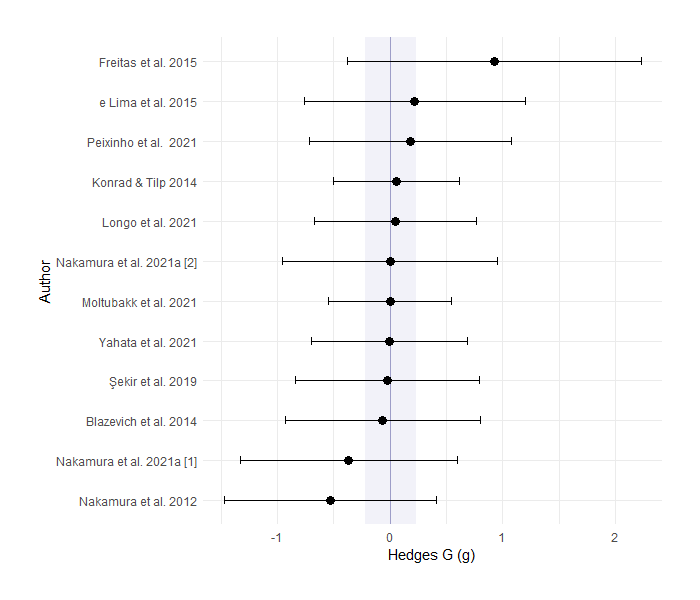


Forest plot for chronic SS studies on ROM


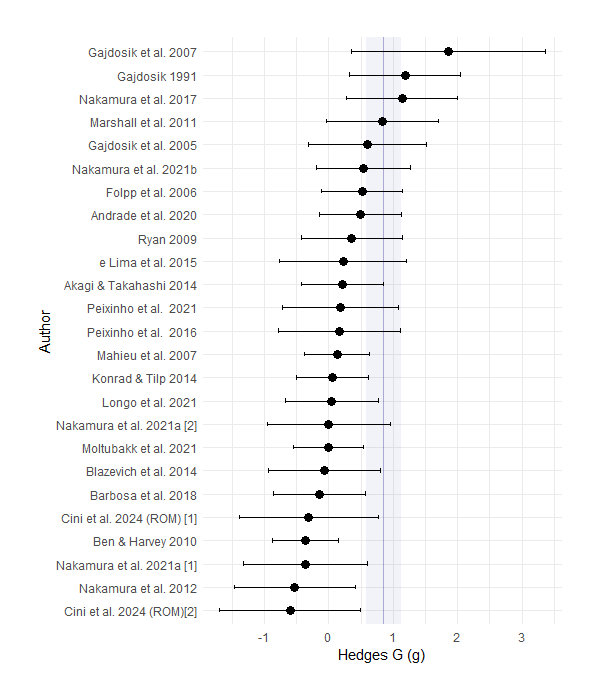

Supplement: Supplementary file 9 — Supplementary file9 (DOCX 6321 KB) [file 40279_2025_2204_MOESM9_ESM.docx]
